# Expert opinion on diagnosing, treating and managing patients with cerebrotendinous xanthomatosis (CTX): a modified Delphi study

**DOI:** 10.1186/s13023-021-01980-5

**Published:** 2021-08-06

**Authors:** Bianca M. L. Stelten, Maria Teresa Dotti, Aad Verrips, Bülent Elibol, Tzipora C. Falik-Zaccai, Kate Hanman, Andrea Mignarri, Belina Sithole, Robert D. Steiner, Surabhi Verma, Gilad Yahalom, Tanyel Zubarioglu, Fanny Mochel, Antonio Federico

**Affiliations:** 1 Department of Neurology, Catharina Hospital, Eindhoven, The Netherlands; 2 Department of Medicine, Surgery and Neurosciences, Medical School, University of Siena and UO Clinical Neurology and Neurometabolic Diseases, AOU Senese, Siena, Italy; 3 Department of Neurology, Canisius-Wilhelmina Hospital, Nijmegen, The Netherlands; 4 Hacettepe University Medical Faculty Hospital, Ankara, Turkey; 5 Institute of Human Genetics, Galilee Medical Center, Naharia, Israel; 6 The Azrieli Faculty of Medicine, Bar Ilan University, Safed, Israel; 7 Costello Medical, London, UK; 8 UO Clinical Neurology and Neurometabolic Diseases, AOU Senese, Siena, Italy; 9 Costello Medical, Manchester, UK; 10 University of Wisconsin School of Medicine and Public Health, Madison, WI USA; 11 Marshfield Clinic Health System, Marshfield, WI USA; 12 Leadiant Biosciences Ltd., London, UK; 13 Shaare Zedek Medical Center, Jerusalem, Israel; 14 Sheba Medical Center, Ramat Gan, Israel; 15 Division of Pediatric Nutrition and Metabolism, Department of Pediatrics, Cerrahpasa Medical Faculty, Istanbul University-Cerrahpasa, Istanbul, Turkey; 16 Reference Center for Adult Neurometabolic Diseases, Department of Genetics, La Pitié-Salpêtrière University Hospital, Paris, France; 17 Department of Medicine, Surgery and Neurosciences, Medical School, University of Siena, Siena, Italy

**Keywords:** Cerebrotendinous xanthomatosis, CTX, Delphi, Diagnosis, Treatment, Prognosis, Monitoring

## Abstract

**Background:**

Cerebrotendinous xanthomatosis (CTX) is a rare, chronic, progressive, neurodegenerative disorder requiring life-long care. Patients with CTX often experience a diagnostic delay. Although early diagnosis and treatment initiation can improve symptoms and prognosis, a standardised approach to diagnosis, treatment and management of patients is not yet established.

**Aim:**

To assess expert opinion on best care practices for patients with CTX using a modified Delphi method.

**Methods:**

A multidisciplinary group of healthcare professionals with expertise in CTX responded to a 3-round online questionnaire (n = 10 in Rounds 1 and 2; n = 9 in Round 3), containing questions relating to the diagnosis, treatment, monitoring, multidisciplinary care and prognosis of patients with CTX. Determination of consensus achievement was based on a pre-defined statistical threshold of ≥ 70% Delphi panellists selecting 1–2 (disagreement) or 5–6 (agreement) for 6-point Likert scale questions, or ≥ 70% Delphi panellists choosing the same option for ranking and proportion questions.

**Results:**

Of the Round 1 (n = 22), Round 2 (n = 32) and Round 3 (n = 26) questions for which consensus was assessed, 59.1%, 21.9% and 3.8% reached consensus, respectively. Consensus agreement that genetic analyses and/or determination of serum cholestanol levels should be used to diagnose CTX, and dried bloodspot testing should facilitate detection in newborns, was reached. Age at diagnosis and early treatment initiation (at birth, where possible) were considered to have the biggest impact on treatment outcomes. All panellists agreed that chenodeoxycholic acid (CDCA) is a lifetime replacement therapy which, if initiated early, can considerably improve prognosis as it may be capable of reversing the pathophysiological process in CTX. No consensus was reached on the value of cholic acid therapy alone. Monitoring patients through testing plasma cholestanol levels and neurologic examination was recommended, although further research regarding monitoring treatment and progression of the disease is required. Neurologists and paediatricians/metabolic specialists were highlighted as key clinicians that should be included in the multidisciplinary team involved in patients’ care.

**Conclusions:**

The results of this study provide a basis for standardisation of care and highlight key areas where further research is needed to inform best practices for the diagnosis, treatment and management of patients with CTX.

**Supplementary Information:**

The online version contains supplementary material available at 10.1186/s13023-021-01980-5.

## Introduction

Cerebrotendinous xanthomatosis (CTX; OMIM 213700) is a rare, autosomal-recessive, lipid storage disease, with more than 400 cases reported worldwide [1–5]. It is caused by pathogenic variants in the *CYP27A1* gene, leading to sterol 27-hydroxylase activity deficiency. This results in reduced primary bile acid synthesis, particularly severely depleted chenodeoxycholic acid (CDCA) levels, abnormal deposition of cholesterol and cholestanol in the tissues, and increased excretion of bile alcohols in urine [2, 3, 6, 7].

CTX is a severe, chronic, progressive disorder requiring life-long care. Several hallmark signs are seen in both paediatric patients (e.g. neonatal cholestatic jaundice and early psychiatric symptoms) [8–11], and occuring throughout life (e.g. infantile-onset chronic diarrhoea, juvenile cataracts, tendon xanthomas, intellectual disability and progressive neurological deterioration) [3, 8]. However, the type, onset and severity of symptoms vary considerably between patients [5, 8].

Alongside the hallmark clinical features, biochemical and molecular genetic tests are typically used to diagnose CTX through detection of increased levels of plasma cholestanol and/or identification of *CYP27A1* pathogenic variants [12]. However, a diagnostic delay of approximately 20–25 years has been reported [3, 8, 13]. This is thought to reflect the difficulty in recognising signs of CTX and lack of awareness surrounding the condition, often leading to misdiagnoses [3]. Increased understanding of best diagnostic practices is therefore needed to facilitate earlier recognition, diagnosis and treatment initiation.

First line treatment uses exogenous CDCA to restore the biochemical abnormalities in CTX [3], thereby improving clinical outcomes. Other licensed and unlicensed therapies that have been used alone or in combination include cholic acid [14], 3-hydroxy-3-methylglutaryl-coenzyme A (HMG-CoA) reductase inhibitors (statins) [14–20], low-density lipoprotein (LDL) apheresis [21–23], and ursodeoxycholic acid (despite not being effective in CTX) [24–28]. Treatment is therefore inconsistent and more specific guidelines on best treatment practices are required. Furthermore, the most appropriate tests for monitoring treatment efficacy and timing for these, is yet to be confirmed [4, 29, 30].

If left untreated, patients experience poorer prognosis, progressive, irreversible neurological damage and reduced life expectancy [14, 31]. Whilst early diagnosis and CDCA treatment initiation can reverse or prevent disease progression/deterioration [10, 31–34], there is still differing opinion about the best time to start treatment, to ensure the best prognosis [3, 35].

There are currently no published guidelines focussing specifically on diagnosis, treatment and management of patients with CTX, despite evidence that early diagnosis and long-term treatment can improve symptoms and prognosis [31–33, 36]. The Delphi method is a systematic and robust methodology that uses iterative rounds of questionnaires to elicit expert consensus opinion [37]. In this modified Delphi panel, conducted between April 2019 and March 2020, we sought to establish consensus on questions regarding best practices for the diagnosis, treatment and management of patients with CTX.

## Methods

### Study design

**Fig. 1:**
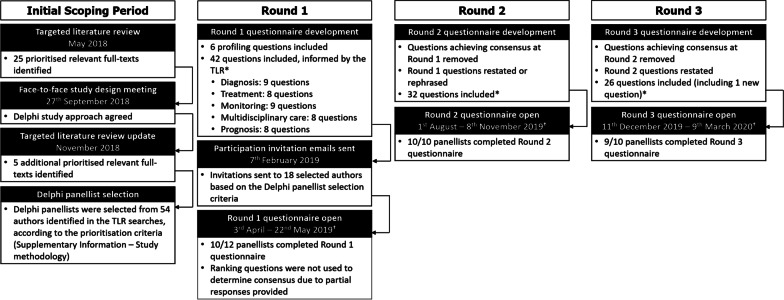
**Delphi study design.** *Total number of questions asked. For some Likert scale questions, multiple options were given, with each analysed separately to assess consensus (e.g. “During the early stages of treatment, paediatric patients should be monitored for the types of symptoms listed below 1–2 times per year. Symptoms: Central and peripheral nervous system; Ocular system; Enterohepatic system; Cognitive performance [e.g. learning difficulties]; Cardiovascular system; Skeletal system; Pulmonary system”, where consensus was assessed separately for each symptom). †For each round, questionnaires were left open for responses in SurveyMonkey until analyses of the results began

This study used a modified Delphi method; whilst classical Delphi studies continue until consensus is achieved for all questions [38], this study included three rounds to avoid questionnaire attrition and to acknowledge that some questions may not reach consensus, even after several rounds (Fig. 1).

To increase the number of experts who could answer questionnaires, an external agency (KH, BSi, AG and DS) independently coordinated the study and developed questionnaires, given the small number of CTX experts worldwide. To ensure relevance, questions were validated by an unpaid expert clinician (BSt) chosen by Leadiant Biosciences, who did not answer questionnaires. Responses remained anonymous to other panellists. Pooled results and individual comments were shared with panellists and Leadiant Biosciences.

### Targeted literature review

A targeted literature review (TLR) was conducted to identify published literature to guide development of the Round 1 questionnaire (Fig. 1). Search terms used in the electronic databases are presented in Additional file 3: Table S1 and full eligibility criteria are shown in Additional file 4: Table S2.

### Delphi panellists

In addition to those who scoped the study design (MTD, AF and AV), experts invited to be panellists were identified based on the first and last authors of relevant full-texts in the TLR, or authors of case studies determined to be relevant at the abstract sift stage of the TLR (Additional file 1: Study methodology). Only the Delphi coordinators and AF, who distributed the email invitations, were aware of panellists’ identities. Invitation emails were sent to 18 potential panellists, with 12 agreeing to participate, 10 completing Rounds 1 and 2, and 9 in Round 3.

### Questionnaire development and distribution

Relevant full-text articles identified from the TLR (Additional file 5: Table S3) were prioritised to inform five sections within the Round 1 questionnaire: diagnosis, treatment, monitoring, multidisciplinary care and prognosis (detailed methodology in Additional file 1: Study methodology and Additional file 2: SurveyMonkey® questionnaires). Questions that reached consensus in Rounds 1 or 2 were removed from the subsequent round. Questions that did not reach consensus were restated or rephrased in the subsequent round to increase the likelihood of achieving consensus (Fig. 1). The Delphi coordinators decided whether to rephrase or restate a question based on the responses in the previous round, comments from the panellists, and the advice of the expert validating the questionnaires (BSt). Round 2 and 3 questionnaires (Additional file 2: SurveyMonkey® questionnaires) were sent alongside individualised Microsoft PowerPoint® (Microsoft, Redmond, Washington) presentations summarising the pooled results of the previous round to encourage elicitation of consensus.

### Question types and pre-specified consensus thresholds

Round 1 included six ‘profiling’ questions to understand panellists’ backgrounds, perspectives and CTX experience (Table 1 and Additional file 2: SurveyMonkey® questionnaires). The five main question types used and pre-specified consensus thresholds, are detailed in Table 1. Each question included “do not wish to answer (DNW)” or “insufficient expertise (IE)” options.

**Table 1:** Question types and consensus thresholds used in questionnaires

Question type	Description	Consensus definition	Delphi questionnaire round
Profiling Questions	Six profiling questions were included to understand the panellists’ background, perspectives and experience of CTX	N/A	N/A
Numeric	Panellists were asked to respond with a single value e.g. *“Please indicate the number of times per year that adult and paediatric patients should be monitored for the types of symptoms described below”*	Not used to determine consensus, but used to inform more specific questions generated for the Round 2 questionnaire	Round 1
Open-Ended	Panellists were asked to provide a free text answer (i.e. there are no options to select from and the panellist can add any response in as much detail as they wish)	Not used to determine consensus, but used to inform more specific questions generated for the Round 2 questionnaire	Round 1
Likert Scale	Panellists were asked to select their level of agreement with a statement e.g. *“Please specify your level of agreement with the following statements by selecting an option from the dropdown list (1–Strongly disagree; 6–Strongly agree)”*	Consensus defined as ≥ 70% of panellists choosing 1–2 (disagreement) or 5–6 (agreement)	Round 1, 2 and 3
Ranking	Panellists were asked to rank the options e.g. *“Please rank these four examinations in order of importance”*	Consensus defined as ≥ 70% of panellists choosing the same ranking position for individual options in the question (e.g. ranking position 1). As a small number of panellists only provided partial responses for ranking questions (i.e. not all options were ranked) in Round 1, ranking questions were not used to determine consensus in this round*	Round 1, 2 and 3
Proportion	Panellists were asked to select the proportion (%) category, that corresponds to the question being asked e.g. *“Please indicate the proportion of paediatric patients that present with the following symptoms, prior to a CTX diagnosis (0–24%; 25–49%; 50–74%; 75–100%)”*	Consensus defined as ≥ 70% of panellists choosing the same proportion (%) category (e.g. 0–24%)	Round 2 and 3

For each question “do not wish to answer (DNW)” or “insufficient expertise (IE)” options were included. *These questions were restated or rephrased in Round 2. Options that reached consensus with respect to a ranking position in Round 2 were removed from Round 3. Panellists were then asked to rank the remaining options in Round 3 using the outstanding ranking positions

CTX: Cerebrotendinous xanthomatosis; N/A: not applicable

### Processing and synthesis of results

Results were exported from SurveyMonkey® and analysed in Microsoft Excel® 2016 (Microsoft, Redmond, Washington). Consensus was assessed by collating and calculating the response distribution. IE responses were excluded in the statistical analyses and DNW was considered neutral (detailed methodology in Additional file 1: Study methodology).

## Results

### Delphi study participation

Of the 12 experts that were sent the Round 1 questionnaire, 10 completed it (Fig. 1). Most panellists were neurologists, with other healthcare professions including geneticists and metabolic specialists, some with paediatric specialities. Most panellists were affiliated to a CTX specialist centre/department, with others from either a local or university hospital, based in a range of countries worldwide (Table 2). The majority had experience of treating > 5 patients with CTX within the past 10 years, with 40% (4/10) having cared for/treated adults only. The number of years for which panellists had been treating patients with CTX, varied (Table 2).

**Table 2:** Panellist demographics

Panellist demographics	Number of panellists, n (%)
*Professional roles of panellists*	
Neurologist	6 (60.0)
Metabolic specialist	1 (10.0)
Geneticist	1 (10.0)
Paediatric metabolic specialist	1 (10.0)
Other*	1 (10.0)
*Place of work of panellists*	
Specialist centre/department	7 (70.0)
Local hospital	2 (20.0)
Other^†^	1 (10.0)
*Country panellists practice in*	
Italy	3 (30.0)
Israel	2 (20.0)
Turkey	2 (20.0)
France	1 (10.0)
The Netherlands	1 (10.0)
USA	1 (10.0)
*Number of patients treated in the past 10 years*	
≥ 21 patients	4 (40.0)
16–20 patients	1 (10.0)
11–15 patients	2 (20.0)
6–10 patients	1 (10.0)
≤ 5 patients	2 (20.0)
*Panellists’ experience in treating adult and paediatric patients*	
Adult patients only	4 (40.0)
Adult and paediatric patients	6 (60.0)
*Years of experience in treating patients with CTX*	
≥ 21 years	3 (30.0)
16–20 years	2 (20.0)
11–15 years	0 (0.0)
6–10 years	3 (30.0)
≤ 5 years	2 (20.0)

Demographics of panellists that responded to the Round 1 questionnaire

^*^‘Other’ response selected and free-text specified as ‘pediatrician, clinical geneticist, and clinical biochemical geneticist (metabolic specialist)’

^†^‘Other’ response selected and free-text specified as ‘university hospital’

CTX: Cerebrotendinous xanthomatosis

### Questionnaire results

In Round 1, of the 22 questions that were used to assess consensus, 13 (59.1%) reached consensus. In Round 2, 32 questions were asked following Round 1 analyses, and 7 (21.9%) of these reached consensus. Following Round 2 analyses, 26 questions were asked in Round 3, with 1 (3.8%) reaching consensus. The results from all three rounds can be found in Table 3 (Likert scale questions), Table 4 (ranking questions) and Table 5 (proportion questions). The response distributions for the Likert scale, ranking and proportion questions can be found in Additional file 6: Tables S4–S6.

**Table 3:** Responses to Likert scale questions

Question	Consensus agreement/disagreement	Percentage agreement/disagreement (%)	Number of panellists responding ‘insufficient expertise’ (n)	Delphi questionnaire round
*Please indicate symptoms that paediatric patients (aged* < 18 years old) present with, prior to a CTX diagnosis*				
Chronic diarrhoea	**Agreement**	**90**	–	Round 1
Bilateral juvenile cataracts	**Agreement**	**90**	–	Round 1
Mental retardation (e.g. learning difficulties)^†^	**Agreement**	**100**	–	Round 1
*Please indicate symptoms that adult patients (aged* ≥ 18 years old) present with, prior to a CTX diagnosis*				
Infantile-onset diarrhoea	**Agreement**	**70**	–	Round 1
Childhood-onset cataracts	**Agreement**	**90**	–	Round 1
Tendon xanthomas	**Agreement**	**90**	–	Round 1
Psychiatric symptoms	**Agreement**	**90**	–	Round 1
Peripheral neuropathy	**Agreement**	**70**	–	Round 1
Cerebellar signs	**Agreement**	**100**	–	Round 1
Pyramidal signs	**Agreement**	**90**	–	Round 1
*All patients have elevated levels of serum cholestanol at the time of diagnosis*	**Agreement**	**100**	–	Round 1
*Brain MRI should be performed at the diagnosis stage as they can contribute to the diagnosis of CTX by revealing abnormally increased or decreased signals with characteristics distribution, but also to exclude other conditions*	**Agreement**	**70**	–	Round 1
*Measurement of serum cholestanol levels is the diagnostic marker of choice for CTX*	**Agreement**	**100**	–	Round 1
*Movement disorders can be considered as late CTX manifestations, however, CTX should be considered in the differential diagnosis of movement disorders, particularly in case of an early onset and when associated with other neurological features and/or with systemic features*	**Agreement**	**70**	–	Round 1
*DBS testing is the optimal method for screening of CTX in newborns*	**Agreement**	**71**	3	Round 1
*CDCA is a lifetime replacement therapy*	**Agreement**	**100**	–	Round 1
*The pathophysiological process in CTX patients may be reversed by CDCA, especially if treatment is initiated early in the disease process*	**Agreement**	**100**	–	Round 1
*Transcranial magnetic stimulation (TMS) is a useful tool for evaluating improvements in pyramidal function in patients receiving CDCA*	**Disagreement**	**71**	3	Round 1
*Treatment adherence can be improved by providing CTX patients with support and intensive education*	**Agreement**	**90**	–	Round 1
*Pre-marital genetic counselling should be recommended to high-risk populations e.g. patients of Israeli or Moroccan origin*	**Agreement**	**75**	2	Round 1
*Please indicate which of the below therapy options improves/stabilises prognosis in the majority of CTX patients*				
CDCA alone	**Agreement**	**100**	–	Round 1
CDCA and HMG-CoA reductase inhibitor	**Agreement**	**78**	1	Round 2
LDL apheresis	**Disagreement**	**71**	3	Round 2
Cholic acid alone	Consensus not reached	Agree (33) Disagree (50)	3	Round 3
Cholic acid and HMG-CoA reductase inhibitor	Consensus not reached	Agree (20) Disagree (60)	4	Round 3
*Reducing plasma cholestanol concentrations slows down the progression of CTX*	**Agreement**	**70**	–	Round 1
*CTX patients who start treatment after significant neurological pathology is established, have a worse prognosis compared to patients who started treatment as early as possible*	**Agreement**	**100**	–	Round 1
*CTX patients showing MRI evidence of cerebellar vacuolation should be monitored more strictly over time as it is considered a prognostic marker*	**Agreement**	**88**	2	Round 1
*During the early stages of treatment, paediatric patients should be monitored for the types of symptoms listed below 1–2 times per year*				
Central and peripheral nervous system	**Agreement**	**100**	1	Round 2
Ocular system	**Agreement**	**78**	1	Round 2
Enterohepatic system	**Agreement**	**89**	1	Round 2
Cognitive performance (e.g. learning difficulties)	**Agreement**	**100**	1	Round 2
Cardiovascular system	Consensus not reached	Agree (44) Disagree (33)	–	Round 3
Skeletal system	Consensus not reached	Agree (67) Disagree (0)	–	Round 3
Pulmonary system	Consensus not reached	Agree (33) Disagree (11)	–	Round 3
*During the early stages of treatment, adult patients should be monitored for the types of symptoms listed below once per year*				
Central and peripheral nervous system	**Agreement**	**100**	–	Round 2
Ocular system	**Agreement**	**70**	–	Round 2
Cardiovascular system	**Agreement**	**70**	–	Round 2
Skeletal system	**Agreement**	**70**	–	Round 2
Enterohepatic system	**Agreement**	**80**	–	Round 2
Cognitive performance (e.g. learning difficulties)	**Agreement**	**100**	–	Round 2
*During the early stages of treatment, adult patients should be monitored for symptoms in the pulmonary system once per year*	Consensus not reached	Agree (38) Disagree (13)	1	Round 3
*Paediatric patients should undergo the types of tests listed below 1–2 times per year*				
Cholestanol plasma concentration	**Agreement**	**78**	1	Round 2
Liver function tests	**Agreement**	**78**	1	Round 2
*Paediatric patients should undergo neurologic (and if necessary neuropsychologic evaluation) testing/examination twice per year*	**Agreement**	**78**	1	Round 2
*Adult patients should undergo the types of tests/examinations listed below once per year*				
Cholestanol plasma concentration	**Agreement**	**90**	–	Round 2
Neurologic (and if necessary neuropsychologic evaluation)	**Agreement**	**100**	–	Round 2
Liver function tests	**Agreement**	**90**	–	Round 2
Urinary bile alcohol concentration	Consensus not reached	Agree (43) Disagree (14)	2	Round 3
Brain MRI	Consensus not reached	Agree (22) Disagree (33)	–	Round 3
*The following healthcare professionals are important in the diagnosis of paediatric patients with CTX*^*‡*^				
Neurologist	**Agreement**	**100**	1	Round 2
Paediatrician/Metabolic specialist	**Agreement**	**89**	1	Round 2
Geneticist	**Agreement**	**78**	1	Round 2
Ophthalmologist	**Agreement**	**100**	–	Round 3
Neuroradiologist	Consensus not reached	Agree (44) Disagree (22)	–	Round 3
Psychiatrist	Consensus not reached	Agree (33) Disagree (0)	–	Round 3
Orthopaedic surgeon	Consensus not reached	Agree (22) Disagree (33)	–	Round 3
Endocrinologist	Consensus not reached	Agree (11) Disagree (67)	–	Round 3
Gastroenterologist	Consensus not reached	Agree (33) Disagree (11)	–	Round 3
*The following healthcare professionals are important in the diagnosis of adult patients with CTX*^*‡*^				
Neurologist	**Agreement**	**100**	–	Round 2
Metabolic specialist	**Agreement**	**80**	–	Round 2
Geneticist	**Agreement**	**80**	–	Round 2
Ophthalmologist	**Agreement**	**78**	–	Round 3
Neuroradiologist	Consensus not reached	Agree (56) Disagree (22)	–	Round 3
Psychiatrist	Consensus not reached	Agree (67) Disagree (0)	–	Round 3
Orthopaedic surgeon	Consensus not reached	Agree (22) Disagree (33)	–	Round 3
Endocrinologist	Consensus not reached	Agree (22) Disagree (56)	–	Round 3
Gastroenterologist	Consensus not reached	Agree (33) Disagree (22)	–	Round 3
Cardiologist	Consensus not reached	Agree (22) Disagree (56)	–	Round 3
*The following healthcare professionals should be involved in prescribing treatment to paediatric patients*^*‡*^				
Neurologist	**Agreement**	**78**	1	Round 2
Neuroradiologist	**Disagreement**	**78**	1	Round 2
Paediatrician/Metabolic specialist	**Agreement**	**89**	1	Round 2
Family doctor	**Disagreement**	**78**	1	Round 2
Endocrinologist	Consensus not reached	Agree (11) Disagree (67)	–	Round 3
Psychiatrist	Consensus not reached	Agree (22) Disagree (44)	–	Round 3
*The following healthcare professionals should be involved in prescribing treatment to adult patients with CTX*^*‡*^				
Neurologist	**Agreement**	**100**	–	Round 2
Neuroradiologist	**Disagreement**	**80**	–	Round 2
Metabolic specialist	**Agreement**	**80**	–	Round 2
Cardiologist	**Disagreement**	**70**	–	Round 2
Family doctor	**Disagreement**	**70**	–	Round 2
Ophthalmologist	**Disagreement**	**70**	–	Round 2
Endocrinologist	**Disagreement**	**78**	–	Round 3
Gastroenterologist	Consensus not reached	Agree (0) Disagree (67)	–	Round 3
Psychiatrist	Consensus not reached	Agree (22) Disagree (44)	–	Round 3
*The following healthcare professionals should be involved in the follow-up of paediatric patients with CTX*^*‡*^				
Neurologist	**Agreement**	**89**	1	Round 2
Paediatrician/Metabolic specialist	**Agreement**	**100**	1	Round 2
Ophthalmologist	**Agreement**	**100**	–	Round 3
Neuroradiologist	Consensus not reached	Agree (33) Disagree (11)	–	Round 3
Family doctor	Consensus not reached	Agree (56) Disagree (0)	–	Round 3
Endocrinologist	Consensus not reached	Agree (22) Disagree (33)	–	Round 3
Gastroenterologist	Consensus not reached	Agree (22) Disagree (11)	–	Round 3
Psychiatrist	Consensus not reached	Agree (44) Disagree (11)	–	Round 3
*The following healthcare professionals should be involved in the follow-up of adult patients with CTX*^*‡*^				
Neurologist	**Agreement**	**100**	–	Round 2
Ophthalmologist	**Agreement**	**70**	–	Round 2
Metabolic specialist	**Agreement**	**80**	–	Round 2
Neuroradiologist	Consensus not reached	Agree (33) Disagree (22)	–	Round 3
Cardiologist	Consensus not reached	Agree (33) Disagree (0)	–	Round 3
Gastroenterologist	Consensus not reached	Agree (33) Disagree (22)	–	Round 3
Family doctor	Consensus not reached	Agree (56) Disagree (0)	–	Round 3
Endocrinologist	Consensus not reached	Agree (33) Disagree (22)	–	Round 3
Psychiatrist	Consensus not reached	Agree (56) Disagree (11)	–	Round 3
*A specialist CTX centre/department should be visited once per year by:*				
Adult patients with CTX	**Agreement**	**100**	–	Round 2
Paediatric patients with CTX	**Agreement**	**89**	1	Round 2
*A local CTX centre/department should be visited twice per year by:*				
Adult patients with CTX	**Agreement**	**90**	–	Round 2
Paediatric patients with CTX	**Agreement**	**100**	1	Round 2
*In patients with CTX, the absence of dentate nuclei signal alteration in brain MRI may be an indicator of better prognosis*	**Agreement**	**75**	2	Round 2
*Increased atrophy and/or signal alteration, identified through brain MRI examinations, may be present in patients who have deteriorating neurological symptoms*	**Agreement**	**78**	1	Round 2
*Research indicates that treating CTX mothers with CDCA during pregnancy acts as an important means of protection against damage to the fetus and miscarriage*	Consensus not reached	Agree (67) Disagree (0)	3	Round 3
*Paediatric patients should undergo testing for urinary bile alcohol concentrations once per year*	Consensus not reached	Agree (57) Disagree (14)	2	Round 3
*Paediatric patients should undergo brain MRI at the time of diagnosis, then once per year during follow-up*	Consensus not reached	Agree (11) Disagree (11)	–	Round 3
*Disease progression in patients with CTX is better monitored using brain MRI compared with clinical evaluation alone*	Consensus not reached	Agree (22) Disagree (44)	–	Round 3
*CDCA alone is a preferred first line treatment compared to CDCA and HMG-CoA reductase inhibitor for treating the underlying biochemical abnormalities in CTX*	**Agreement**	**78**	–	Round 3
*There is a positive correlation between the progression of clinical and neuroradiological symptoms in patients with CTX*	Consensus not reached	Agree (50) Disagree (0)	1	Round 3
*Brain MRI can be used to determine neurological stability in patients with CTX*	Consensus not reached	Agree (25) Disagree (25)	1	Round 3

A total of 10 panellists answered questions in Rounds 1 and 2, and 9 in Round 3. Questions achieving consensus (≥ 70% panellists agreeing/disagreeing with the statement) are shown for the round in which consensus was reached and are highlighted in **bold**. Where questions did not achieve consensus throughout the study, the results are shown for Round 3 only. The proportion of panellists responding neutrally to the questions (responding ‘3’,‘4’ or ‘DNW’) are not presented. In all rounds, ‘insufficient expertise’ responses were removed prior to analysis. *Options that did not achieve consensus in Round 1 were rephrased as a proportion question in Round 2. Please refer to Table 5 for the rephrased questions and responses

^†^Phrased as in the original survey question; ‘mental retardation’ referred to as ‘intellectual disability’ in the text

^‡^Some panellists noted in their comments that alternative roles/names for certain healthcare specialists exist in different countries (e.g. metabolic specialists are often the same as geneticists in some countries). Therefore, panellists may have answered questions about healthcare professionals differently depending on the role they perceive each of the healthcare professionals’ specialism to be in their country

CDCA: chenodeoxycholic acid; CTX: Cerebrotendinous xanthomatosis; DBS: dried bloodspot; DNW: do not wish to answer; HMG-CoA: 5-hydroxy-3-methylglutaryl-coenzyme A; LDL: low-density lipoprotein; MRI: magnetic resonance imaging; TMS: transcranial magnetic stimulation

**Table 4:** Responses to ranking questions

Question	Ranking position that reached consensus	Rank selected (% selecting specified rank)	Delphi questionnaire round
*Please rank the following indicators in order of which has the greatest diagnostic value, when considering a CTX diagnosis (1* = *greatest diagnostic value; 5* = *least diagnostic value)*			
*CYP27A1* genetic mutation*	**1**	**1 (80)**	Round 2
An affected sibling	Consensus not reached	2 (33) 3 (33) 4 (0) 5 (33)	Round 3
Clinical signs and symptoms	Consensus not reached	2 (22) 3 (33) 4(44) 5 (0)	Round 3
Biochemical pathogenesis	Consensus not reached	2 (56) 3 (22) 4 (11) 5 (11)	Round 3
Brain MRI findings	Consensus not reached	2 (0) 3 (22) 4 (22) 5 (56)	Round 3
*Please rank the following tests/examinations in order of importance when confirming a CTX diagnosis (1* = *most important; 5* = *least important)*			
Genetic testing alone	**1**	**1 (90)**	Round 2
Determination of serum cholestanol levels	**2**	**2 (80)**	
Detection of urinary bile alcohols	Consensus not reached	3 (38) 4 (50) 5 (13)	Round 3
Determination of plasma bile acids (mainly cholic acid and chenodeoxycholic acid)	Consensus not reached	3 (33) 4 (56) 5 (11)	Round 3
Conventional brain MRI	Consensus not reached	3 (22) 4 (33) 5 (44)	Round 3
*Please rank the following factors in order of their impact on treatment outcomes in patients with CTX (1* = *greatest impact; 5* = *least impact)*			
Age at diagnosis and treatment initiation	**1**	**1 (90)**	Round 2
Extent of neurological deterioration	**2**	**2 (80)**	Round 2
Cholestanol level at diagnosis	**5**	**5 (89)**	Round 3
Treatment compliance	Consensus not reached	3 (67) 4 (22) 5 (11)	Round 3
Characteristics of cerebellar signal abnormalities	Consensus not reached	3 (33) 4 (67) 5 (0)	Round 3
*Please rank the following therapy options in order of their effectiveness for treating the underlying biochemical abnormalities in CTX (1* = *most effective; 5* = *least effective)*			
CDCA alone	**1**	**1 (80)**	Round 2
LDL apheresis	**5**	**5 (71)**	Round 2
CDCA and HMG-CoA reductase inhibitor^†^	**2**	**2 (71)**	Round 3
Cholic acid alone	Consensus not reached	2 (33) 3 (0) 4 (67)	Round 3
Cholic acid and HMG-CoA reductase inhibitor	Consensus not reached	2 (20) 3 (60) 4 (20)	Round 3
*Please indicate when the most beneficial time to start CTX treatment is by ranking the below options (1* = *most beneficial; 4* = *least beneficial)*			
From birth following a positive newborn screening test for CTX	**1**	**1 (90)**	Round 2
Upon CTX diagnosis (with or without symptom onset)	**2**	**2 (80)**	Round 2
Upon symptom onset in patients diagnosed with CTX	**3**	**3 (90)**	Round 2
Upon presentation of neurological symptoms in patients diagnosed with CTX	**4**	**4 (90)**	Round 2
*Please rank the following examinations and tests in order of their usefulness when monitoring paediatric patients receiving CTX treatment (1* = *most useful; 5* = *least useful)*			
Cholestanol plasma concentration	**1**	**1 (78)**	Round 2
Neurologic examination (and if necessary neuropsychologic evaluation)	**2**	**2 (78)**	Round 3
Brain MRI	Consensus not reached	2 (11) 3 (33) 4 (33) 5 (22)	Round 3
Liver function tests	Consensus not reached	2 (22) 3 (44) 4 (11) 5 (22)	Round 3
Urinary bile alcohol concentration	Consensus not reached	2 (13) 3 (13) 4 (50) 5 (25)	Round 3
*Please rank the following examinations and tests in order of their usefulness when monitoring adult patients receiving CTX treatment (1* = *most useful; 5* = *least useful)*			
Cholestanol plasma concentration	**1**	**1 (70)**	Round 2
Neurologic examination (and if necessary neuropsychologic evaluation)	**2**	**2 (78)**	Round 3
Brain MRI	Consensus not reached	2 (11) 3 (33) 4 (44) 5 (11)	Round 3
Liver function tests	Consensus not reached	2 (22) 3 (44) 4 (11) 5 (22)	Round 3
Urinary bile alcohol concentration	Consensus not reached	2 (13) 3 (0) 4 (50) 5 (38)	Round 3
Levels of serum cholestanol alone	Consensus not reached	1 (22) 2 (22) 3 (22) 4 (22) 5 (11)	Round 3
Clinical presentation/neurological examination	Consensus not reached	1 (56) 2 (22) 3 (11) 4 (0) 5 (11)	Round 3
Brain MRI	Consensus not reached	1 (0) 2 (11) 3 (44) 4 (22) 5 (22)	Round 3
Levels of urinary bile alcohols	Consensus not reached	1 (13) 2 (0) 3 (25) 4 (38) 5 (25)	Round 3
Electrophysiological examinations (e.g. electromyography, nerve conduction velocity, electroencephalography)	Consensus not reached	1 (11) 2 (22) 3 (22) 4 (11) 5 (33)	Round 3

A total of 10 panellists answered questions in Rounds 1 and 2, and 9 in Round 3. Ranking positions achieving consensus (≥ 70% panellists ranking an option in a particular position) are shown for the round in which consensus was reached and highlighted in **bold**. Where questions did not achieve consensus throughout the study, the results are shown for Round 3. In some cases, panellists assigned the same ranking position to multiple options. If consensus on a ranking position was achieved in Round 2, panellists were not asked to rank options in that position in Round 3. *Phrased as in the original survey question; ‘genetic mutations’ referred to as ‘pathogenic variants’ in the text

^†^Panellists came to consensus agreement about CDCA alone in Round 1 and CDCA in combination with HMG-CoA reductase inhibitors in Round 2, where CDCA alone was no longer included as an option

CDCA: chenodeoxycholic acid; CTX: Cerebrotendinous xanthomatosis; HMG-CoA: 5-hydroxy-3-methylglutaryl-coenzyme A; LDL: low-density lipoprotein; MRI: magnetic resonance imaging

**Table 5:** Responses to proportion questions

Question	Proportion that reached consensus	Proportion selected (% selecting specified proportion)	Delphi questionnaire round
*Please indicate the proportion of paediatric patients that present with the following symptoms, prior to a CTX diagnosis*			
Tendon xanthomas	**0–24%**	**0–24% (89)**	Round 3
Early psychiatric symptoms (e.g. autism)	Consensus not reached	0–24% (33) 25–49% (56) 50–74% (11) 75–100% (0)	Round 3
Neonatal cholestatic jaundice	Consensus not reached	0–24% (38) 25–49% (38) 50–74% (25) 75–100% (0)	Round 3
Cerebellar system findings (e.g. ataxia symptoms and tremor)	Consensus not reached	0–24% (33) 25–49% (22) 50–74% (33) 75–100% (11)	Round 3
Epilepsy	Consensus not reached	0–24% (33) 25–49% (56) 50–74% (11) 75–100% (0)	Round 3
Peripheral neuropathy*	Consensus not reached	0–24% (56) 25–49% (11) 50–74% (33) 75–100% (0)	Round 3
Z-scores below the expected range for age in bone mineral density (BMD)*	Consensus not reached	0–24% (67) 25–49% (17) 50–74% (17) 75–100% (0)	Round 3
*Please indicate the proportion of adult patients that present with the following symptoms, prior to a CTX diagnosis*			
Early-onset dementia	**25–49%**	**25–49% (70)**	Round 2
Early-onset movement disorder (e.g. atypical parkinsonism)	Consensus not reached	0–24% (44) 25–49% (44) 50–74% (0) 75–100% (11)	Round 3
Epilepsy	Consensus not reached	0–24% (56) 25–49% (33) 50–74% (11) 75 –100% (0)	Round 3

A total of 10 panellists answered questions in Rounds 1 and 2, and 9 in Round 3. Options achieving consensus (≥ 70% panellists selecting a particular proportion for that option) are shown for the round in which consensus was reached and highlighted in **bold**. Where questions did not achieve consensus throughout the study, the results are shown for Round 3. In some cases, panellists selected the same proportion for different options. *In Round 2 these options were phrased in one option as ‘Peripheral neuropathy where Z-scores are below the expected range for age in bone mineral density (BMD)’, however, for scientific accuracy it was decided to split this into two options in Round 3. BMD: bone mineral density; CTX: Cerebrotendinous xanthomatosis

#### Diagnosis

Panellists agreed that symptoms presented by paediatric patients prior to a CTX diagnosis include chronic diarrhoea, bilateral juvenile cataracts and intellectual disability (e.g. learning difficulties). Panellists agreed that symptoms presented by adults prior to diagnosis are infantile-onset diarrhoea, childhood-onset cataracts, tendon xanthomas, psychiatric symptoms and neurological symptoms (peripheral neuropathy, cerebellar and pyramidal signs). Panellists also agreed that early-onset dementia presents in 25–49% of adults before diagnosis. It was agreed that movement disorders can be late CTX manifestations, however, CTX should be considered in the differential diagnosis of movement disorders, particularly in early-onset cases and when associated with other neurological and/or systemic features.

In terms of the most important indicator when considering a CTX diagnosis, *CYP27A1* pathogenic variants were considered to be of greatest diagnostic value. When ranking the importance of tests/examinations used to confirm a CTX diagnosis, panellists agreed that genetic testing is most important, followed by determination of serum cholestanol levels. All panellists responded that patients always have elevated serum cholestanol at diagnosis and that measuring serum cholestanol is the diagnostic marker of choice. It was agreed that dried bloodspot (DBS) testing is the optimal method for screening of CTX in newborns.

#### Treatment

Panellists agreed that the most beneficial time to start CTX treatment is from birth, following a positive newborn screening test. Initiating treatment upon CTX diagnosis (with or without symptom onset) was agreed to be the next most beneficial option, followed by starting treatment upon symptom onset in diagnosed patients.

All panellists agreed that CDCA is a lifetime replacement therapy which may be capable of reversing the pathophysiological process in CTX, especially if initiated early in the disease process. The following ranking order was agreed upon when panellists were asked to consider therapy options that are effective for treating the underlying biochemical abnormalities in CTX: CDCA alone was ranked first, CDCA and HMG-CoA reductase inhibitor together was ranked second, and LDL apheresis was ranked last. No consensus was reached for other treatment options such as cholic acid alone.

Consensus was not reached on how useful available parameters for measuring treatment efficacy in patients with CTX are. In Round 1, serum cholestanol levels and clinical presentation/neurological examination were ranked as most useful by 30% and 50% of panellists respectively. In Round 2, serum cholestanol levels alone was most commonly ranked as the most useful parameter (50% of panellists agreed), whilst in Round 3, clinical presentation/neurological examination was most commonly ranked first (56% of panellists agreed). However, brain MRI was not ranked the most useful parameter for measuring treatment efficacy in any round.

The following order was agreed upon when panellists were asked to consider factors that have the greatest impact on treatment outcomes: age at diagnosis and treatment initiation was ranked first, the extent of neurological deterioration was ranked second and cholestanol levels at diagnosis was ranked last.

#### Monitoring

Panellists agreed that providing patients with support and intensive education improves treatment adherence. Panellists were in consensus disagreement that transcranial magnetic stimulation (TMS) is a useful tool for evaluating pyramidal function improvements in patients receiving CDCA. When asked about monitoring symptoms (during early stages of treatment as this is when dose adjustment may be necessary), panellists agreed that cognitive development and symptoms of the ocular, enterohepatic and central/peripheral nervous systems should be monitored in paediatric patients once or twice annually, and in adults annually. Additionally, symptoms of the cardiovascular/skeletal systems should be monitored in adults annually.

The following order was agreed upon when panellists were asked which examinations/tests for monitoring paediatric and adult patients receiving treatment are most useful: testing for cholestanol plasma concentration was ranked first and neurologic examination (and if necessary neuropsychologic examination) was ranked second. No consensus was reached on the ranking order for brain MRI, liver function tests and urinary bile alcohol concentration.

Panellists agreed that paediatric patients should undergo tests for plasma cholestanol concentration and liver functions once or twice annually, and neurologic (and if necessary neuropsychologic evaluation) examination twice annually. It was agreed that adults should undergo each of these tests once annually. Regarding the use of brain MRI for monitoring patients, there was no consensus on whether patients should have an MRI once annually. Comparing brain MRI and clinical evaluation, consensus was not reached on whether MRIs allow tracking of CTX disease progression with greater sensitivity than clinical scales and whether they should be used during follow-up.

#### Multidisciplinary care

Panellists agreed that neurologists and paediatricians/metabolic specialists should be involved in the diagnosis of, prescribing treatment to and follow-up of all patients with CTX. Agreement regarding the roles of other clinicians is presented in Table 3.

#### Prognosis

All panellists agreed that patients with CTX who start treatment after significant neurological pathology is established, have a worse prognosis compared to those who start treatment as early as possible. All experts responded that CDCA alone improves/stabilises prognosis in the majority of patients with CTX. When considering CDCA in combination with HMG-CoA reductase inhibitor without being presented with an option of CDCA alone, panellists agreed that this therapy option also improves/stabilises prognosis. However, panellists responded that CDCA alone is the preferred first line treatment compared to CDCA in combination with HMG-CoA reductase inhibitor for treating underlying biochemical abnormalities in CTX. Panellists disagreed that LDL apheresis improves/stabilises prognosis. No consensus was reached on whether therapy with cholic acid alone or in combination with HMG-CoA reductase inhibitor improves/stabilises prognosis. It was agreed that reducing plasma cholestanol concentrations slows down CTX progression.

Consensus was reached on some questions regarding the use of neurological imaging to indicate disease prognosis. Panellists agreed that patients showing MRI evidence of cerebellar vacuolation should be monitored more strictly over time as it is considered a poor prognostic marker. There was agreement that increased atrophy and/or signal alteration identified through brain MRIs may be present in those with deteriorating neurological symptoms. Additionally, panellists agreed that the absence of dentate nuclei signal alteration may indicate better prognosis. Consensus was not reached on whether brain MRI can be used to determine neurological stability.

## Discussion

There are currently no standard guidelines on the diagnosis, treatment and management of patients with CTX. This Delphi study achieved consensus on aspects of care for paediatric and adult patients, from a group of experts.

In paediatric patients, chronic diarrhoea, bilateral juvenile cataracts and intellectual disability (e.g. learning difficulties) were identified as key symptoms, supported by available literature [3, 9]. For patients who are not diagnosed until adulthood, additional symptoms include tendon xanthomas, psychiatric and neurological symptoms. Presence of these signs should prompt clinicians to refer patients for further testing, and greater awareness of these typical symptoms may aid early diagnosis and treatment [3]. Whilst some signs asked about in these questions did not reach consensus, in some instances their presentation should still prompt further investigation. As clinicians may only see a small subset of patients, whose symptoms will likely vary from patients seen by other physicians due to the heterogenous nature of CTX, this may indicate why consensus was not achieved for these questions. For example, neonatal cholestatic jaundice can often be self-limiting [39, 40], and so the proportion of patients presenting to each expert with this symptom could vary, dependant on the age of patients at presentation. Nevertheless, when prolonged and without a specific diagnosis, neonatal jaundice should raise suspicion towards a CTX diagnosis given it can potentially cause irreversible liver damage if left untreated [8, 41].

Presence of biallelic *CYP27A1* pathogenic variants was considered to be the indicator of greatest diagnostic value. This suggests that, whilst the presence of key symptoms may prompt further investigation [3], molecular analysis of the *CYP27A1* gene should be considered the primary means for diagnosis, with whole exome sequencing showing great promise for increasing accurate diagnoses [42]. Results indicate that patients always have elevated serum cholestanol levels and that this should be considered an appropriate secondary means for investigating a diagnosis. However, the fact that in some atypical cases patients exhibit normal cholestanol levels [43], should be acknowledged. Furthermore, raised serum cholestanol levels have been described on occasion in patients with primary biliary cirrhosis and Niemann Pick type C, and very rarely in progressive familial intrahepatic cholestasis type 3 [44]. The fact that elevated cholestanol levels are sometimes observed in other conditions should therefore be recognised when considering this indicator in the investigation of a CTX diagnosis.

An alternative means for diagnosis, suggested as the solution for early identification of patients with CTX, is through newborn screening [45]. Several biomarkers have been described, with 5β-cholestane-3α,7α,12α,25-tetrol glucuronide (GlcA-tetrol) and the ratio of GlcA-tetrol to tauro-chenodeoxycholic acid (GlcA-tetrol/t-CDCA), highlighted as key candidate biomarkers for newborn screening [46, 47]. However, no national programme has yet been implemented [4]. It was agreed in this study that DBS testing is the optimal method for newborn screening and that starting treatment from birth, following a positive newborn screening test, is most beneficial. Supported by agreement that age at diagnosis and treatment initiation has the biggest impact on treatment outcomes, this indicates that suggestions for pilot screening studies should be taken forward [45]. However, this study did not explore the most appropriate biomarkers for CTX screening in newborns and so further research is needed to assess the best options for potential future newborn screening programmes [46].

Panellists agreed that providing support and intensive education to patients with CTX can improve treatment adherence, and that pre-marital genetic counselling should be recommended to high-risk populations. Such populations could, for example, include Moroccan Jews and the Druze community in the Middle East [48, 49]. Given the relatively high frequency of autosomal recessive diseases in these populations [50], the feasibility of providing counselling to these patients would need to be carefully considered.

There was agreement that CDCA is a lifetime replacement therapy that could reverse the pathophysiological process in CTX and improve/stabilise prognosis, and that it is the most effective therapeutic option available for treating underlying biochemical abnormalities, in line with recommendations in England that CDCA should be used in CTX treatment [51, 52]. Whilst panellists agreed that combination therapy with CDCA and HMG-CoA reductase inhibitor is also effective for treating CTX, CDCA alone was the preferred first line treatment. This aligns with literature where CDCA is considered to be standard of care for CTX [3, 33, 45]. Conversely, the lack of consensus about the effectiveness of cholic acid aligns with the paucity of evidence in the literature regarding its safety and efficacy, indicating there is not sufficient support for its routine use in CTX [4]. This is despite its recommendation in England as a second line treatment when CDCA is no longer tolerated or effective, and indication for bile acid synthesis disorders due to single enzyme defects including CTX in the USA [52, 53]. The consensus in this study that LDL apheresis is the least effective therapy option indicates that its use in CTX may not be appropriate.

Consensus was not reached on methods for measuring treatment efficacy. However, panellists agreed that cholestanol plasma concentration tests are most useful for monitoring treatment over time (for example, in facilitating dose changes), and should occur one to two times annually for paediatric patients and once annually for adults. Furthermore, panellists agreed that reducing plasma cholestanol concentrations slows down CTX progression. However, while serum cholestanol may be useful for evaluating treatment compliance [3], research on the impact of cholestanol levels, and whether cumulative cholestanol exposure correlates to disease progression, is lacking. Further research is needed to collect data that determine the usefulness of plasma cholestanol levels, or other metabolic precursors, for monitoring CTX.

This study emphasises the importance of initiating treatment early [32, 34]; if possible, prior to presentation of neurological symptoms, to ensure better prognosis. There was consensus surrounding neurological signs that should be monitored to determine prognosis; panellists agreed that evidence of cerebellar vacuolation, increased atrophy and/or signal alteration in brain MRIs can suggest worsening prognosis, whilst absence of dentate nuclei signal alteration in MRIs could indicate better prognosis. However, it is not clear what tools should be used for monitoring neurological signs, as demonstrated by the lack of consensus around some related questions in this study. Panellists did, however, agree that liver function tests and neurologic examination should be used to monitor all patients.

As a technique for eliciting expert consensus, the Delphi method has several advantages, allowing a variety of opinions to be gathered from a heterogenous sample of experts and for questionnaires to develop based on results and feedback in free-text responses. In our study, anonymity of panellists and their responses, and the use of an independent agency to coordinate the study, minimised external bias and maximised expert participation, as they did not have to act as coordinators. Validation of questions by one clinical expert who did not respond to the questionnaires ensured they were of high relevance and accuracy, maximising the usefulness of the output toward recommendations for the care of patients. Use of an online tool allowed responses to be quickly gathered from a group of geographically dispersed experts, and rapidly analysed.

However, there are some limitations to the Delphi method. Unlike standard questionnaires, Delphi studies require ongoing time commitment from panellists which can lead to questionnaire attrition. We minimised this by designing the study to only have 3 rounds, thereby not requiring panellists to commit to additional rounds if questions did not reach consensus, whilst still ensuring that all questions not achieving consensus were asked at least twice. There was therefore high panellist retention, with only one panellist dropping-out after Round 2. The Delphi study format also means that questions are interpreted by panellists without any explanation further to what is provided. However, having multiple survey rounds where panellists receive the pooled results from the previous round reduces the possible bias introduced through misinterpretation of questions, by providing an opportunity for re-interpretation in line with the group’s responses. Nevertheless, profiling questions, or questions that achieved consensus in Round 1, could have been subject to varied interpretation, presenting a possible limitation to this methodology. Furthermore, research indicates that social-psychological factors can cause experts with divergent views to feel pressure to conform [38]. Whilst AF, MD and AV received honoraria to attend the initial face-to-face study design meeting in September 2018, to limit bias introduced through sponsorship of this study by industry, panellists did not receive funding for participation in the Delphi panel. All panellists accepted authorship following completion of the study.

Given that CTX is a rare disease and there are consequently few experts worldwide, only a relatively small number of experts participated (n = 10 for Rounds 1 and 2; n = 9 for Round 3), representing a limitation of the study. Another limitiation of the study is the geographical spread of the panel, which was restricted as few experts worldwide from a relatively limited number of specialist centres were eligible to/agreed to participate. A larger and more diverse panel would ensure greater robustness and representativeness of the CTX population, although including experts from Europe, North America and Asia mitigated the limitation to some extent. A further limitation is that some experts had not been treating patients for very long and had treated relatively few patients in the past 10 years, which may be reflective of the small and dispersed population of patients with CTX, as a rare disease.

This study highlights several areas where expert opinion is aligned on the diagnosis, treatment and management of patients with CTX. However, there were some areas where consensus was not achieved; reasons that Delphi studies may not achieve higher consensus levels can include small sample sizes, divided clinical opinion, variability in disease presentation and importantly, a lack of data on particular topics being assessed. This study demonstrated that techniques for monitoring treatment efficacy and CTX progression require further investigation. In particular, given the importance of monitoring progression of neurological symptoms, further research to inform guidelines could be undertaken. Whilst the value of MRI biomarkers toward revealing disease prognosis was indicated, their use for measuring clinical improvement compared with clinical scales requires further investigation, e.g. benefits during presymptomatic stages or during slow disease evolution [29]. Further research also needs to identify what follow-up tests/examinations are appropriate based on the heterogenous clinical presentation of patients. Whilst offering a preliminary insight into preferred approaches for the care of patients with CTX, further data need to be collected to substantiate these findings, fill outstanding knowledge gaps, and inform best practices. Furthermore, this study collected opinions from healthcare professionals exclusively, and future research should therefore look to understand the impact of these decisions on patient/carer quality of life by gathering their opinions. Additionally, in light of agreement that provision of support and intensive education to patients can improve treatment adherence, opportunities to build on this in the future could be explored.

## Conclusion

This Delphi study elicited consensus expert opinion on a number of factors relating to the diagnosis, treatment and management of patients with CTX. Results showed that, with a wide variety of symptoms throughout patients’ lifetimes, prompt diagnosis should be facilitated using genetic analyses or determination of serum cholestanol levels, or screening via DBS testing in newborns. Age at diagnosis and beginning treatment early (at birth, where possible) were considered to have the biggest impact on treatment outcomes and panellists agreed that early initiation of lifetime CDCA replacement therapy may considerably improve prognosis. No consensus was reached on the value of cholic acid therapy alone. Whilst results showed that patients should be monitored through plasma cholestanol concentration testing and neurologic examination once or twice annually, further research is needed regarding monitoring treatment/progression of the disease. This study highlights where further data are needed to inform best practices and provides an indication of preferred approaches for the care of patients with CTX.

## Supplementary Information

**Additional file 1**. Study methodology.

**Additional file 2**. SurveyMonkey® questionnaires.

**Additional file 3. Table S1** - TLR search terms.

**Additional file 4. Table S2** - TLR eligibility criteria.

**Additional file 5. Table S3** - Full-texts prioritised from the TLR to inform Delphi panel questionnaires.

**Additional file 6. Tables S4–S6** - Response distributions.

## Data Availability

The datasets used and/or analysed during the current study are available from the corresponding author on reasonable request.
