# Supplementary material for: Expert opinion on diagnosing, treating and managing patients with cerebrotendinous xanthomatosis (CTX): a modified Delphi study
Source: Orphanet J Rare Dis. 2021 Aug 6;16:353. doi: 10.1186/s13023-021-01980-5 (PMC8349076; doi:10.1186/s13023-021-01980-5)
Supplement: Supplementary file 1 — Additional file 1. Study methodology. [file 13023_2021_1980_MOESM1_ESM.pdf]

Supplementary Information – Study methodology

Study Design

A face-to-face meeting was held in September 2018 with three CTX clinical experts (MTD, AF and AV; selected by Leadiant Biosciences due to their position as distinguished CTX experts in the field), to decide the approach for the Delphi panel study (Figure 1).

Targeted Literature Review

In both the TLR (May 2018) and TLR update (November 2018), used to guide development of the Round 1 questionnaire, case studies/reports were included in a separate group to inform Delphi panellist selection, but were not taken forward to the full-text stage. Instead, review papers and articles on broader studies were prioritised to enable the identification of key topics in the care of patients with CTX, rather than focussing on the experiences of individual patients through case studies/reports.

Delphi Panellist Selection

The TLR was used to identify experts eligible to be invited as panellists in the study. Eligible experts included authors of any relevant full-texts or authors of any case studies determined to be relevant at the abstract sift stage of the TLR. The latter was included as it was recognised that some key experts may only have published case studies/reports, rather than the review papers and articles on broader studies taken through to full-text stage. As many authors were identified, further criteria were used to determine who should be invited to participate in the study. Authors on articles and case studies dated before 1996 were excluded and only the first and last authors of the remaining articles were eligible (n=54). Clinical specialisms and geographical locations for the 54 identified authors were searched. Based on these findings and an update to the TLR, working in collaboration with Leadiant Biosciences, the Delphi panel coordinators then selected the group of experts to be invited

to participate in the study, ensuring that a range of specialisms and geographies were included.

To maximise the likelihood of recruitment, authors were invited to participate in the study via emails sent by AF, which were prepared by the Delphi coordinators. Invited experts also had the opportunity to recommend additional experts that could be invited as panellists. This helped to ensure that any relevant experts who may not have been captured in the TLR were invited.

Invitations were sent to 18 authors based on the selection criteria. Although 12 agreed to participate, only 10 completed the questionnaire. Having received the questionnaire, one expert indicated that they did not feel they had the right expertise to participate, whilst the reason for the other not participating was unknown.

Development and Distribution of the Questionnaires

As a large number of relevant full-texts were identified in the TLR and subsequent update, Leadiant Biosciences and the Delphi coordinators prioritised 30 articles which were used to inform the Round 1 questionnaire. Prioritised articles covered a wide range of geographical locations, to reduce bias, and were mainly review papers (Supplementary Table 3). Questionnaires were delivered to panellists through SurveyMonkey® (SurveyMonkey Inc., San Mateo, California, USA, www.surveymonkey.com) using a weblink via email. Questionnaires for each round were left open until analyses for that round were initiated. The abstracts of all relevant full-texts (n=86) identified in the TLR and subsequent update were presented to panellists in an abstract book alongside the Round 1 questionnaire, for their reference. The abstracts of the 30 prioritised full-texts used to inform the Round 1 questionnaire were clearly indicated in the abstract book. Individualised Microsoft PowerPoint® (Microsoft, Redmond, Washington) presentations were sent to each panellist alongside the Round 2 and 3 questionnaires (Supplementary Information 3 and

Supplementary Information 4, respectively). The individualised presentations contained the pooled results and a reminder of the panellist's individual response to each question. Only the Delphi coordinators viewed individual panellists' responses to questions.

Question Types and Pre-Specified Consensus Thresholds

Likert scale questions not achieving consensus in Round 1 were restated or rephrased in Round 2. Questions that did not achieve consensus in Round 2 were restated in Round 3 to ensure each question had been asked twice in the same format. In some instances, options that did not achieve consensus using Likert scale questions in Round 1 were rephrased as proportion-type questions in Round 2.

Ranking questions were not used to obtain consensus in Round 1 as they were only partially completed by some panellists. Instead, they were rephrased or restated in Rounds 2 and 3. Options that reached consensus with respect to a ranking position in Round 2 were removed from Round 3. Panellists were then asked to rank the remaining options in Round 3 using the outstanding ranking positions. For ranking questions in Rounds 2 and 3, some panellists ranked more than one option at the same level.

Numeric questions were not used to obtain consensus in Round 1 and were used to inform Likert scale question types in Rounds 2 and 3.

Consensus was not assessed for open-ended questions in Round 1; rather, responses were used to gather ideas from panellists and generate more specific questions in Round 2.

Processing and Synthesis of Results

In Likert scale question analyses, DNW was considered neutral (i.e. the respondent neither agrees nor disagrees; Table 1). For ranking and proportion questions, respondents selecting DNW were considered to have not selected the ranking option being analysed but were

included in the total number of participants who answered that question. No respondents selected DNW for numeric questions.
