# Supplementary material for: Expert opinion on diagnosing, treating and managing patients with cerebrotendinous xanthomatosis (CTX): a modified Delphi study
Source: Orphanet J Rare Dis. 2021 Aug 6;16:353. doi: 10.1186/s13023-021-01980-5 (PMC8349076; doi:10.1186/s13023-021-01980-5)
Supplement: Supplementary file 2 — Additional file 2. SurveyMonkey® questionnaires. [file 13023_2021_1980_MOESM2_ESM.pdf]

SurveyMonkey® questionnaires

Round 1 Questionnaire

First Round CTX Delphi Panel Questionnaire
Introduction

Thank you for participating in this Delphi panel and for taking the time to complete this Round 1 questionnaire. The objective of the Delphi panel is to achieve consensus on best practices for diagnosing, treating and managing patients with cerebrotendinous xanthomatosis (CTX).

Delphi Panel Methodology

The Delphi method is a technique that is often used to gather consensus on specific topics from a group of experts, by conducting a series of questionnaires.[1] The method utilises an iterative process where results from the previous round are reported to participants, to provide them with an opportunity to reassess their initial judgements on the information in questions.[2, 3] The Delphi method is characterised by multiple rounds of questionnaires, participant anonymity and the controlled feedback process. Responses are assessed based on whether they reach the pre-defined consensus threshold.

The consensus threshold for this study has been set to 70% agreement or disagreement. For further details of how the consensus threshold will be applied to different question types please see Appendix 1.

Three rounds of questionnaires will be used in this Delphi panel, all of which will be completed remotely using an online platform.

Questionnaire Development and Pre-Reading

A targeted literature review (TLR) was conducted to identify published literature to guide the development of this questionnaire. The purpose of the TLR was to identify relevant literature relating to the diagnosis, treatment, monitoring, multidisciplinary care and prognosis of CTX patients.

The citations of all relevant abstracts identified in the TLR have been provided alongside this questionnaire as pre-reading material. Additionally, 30 of these citations have been prioritised as key articles which have informed the questions. The citations for the 30 articles are shown in Appendix 2 and the abstracts are presented in the pre-reading material that accompanies this questionnaire. Please note that the optional pre-reading material is provided for reference only and as a participant you are not obliged to read these before completing the questionnaire.

Questionnaire Structure and Format

The first round of the questionnaire contains six profiling questions, of which the responses will help us to understand your background and also your perspective and experience of CTX. Please note that your answers to the profiling questions will not be shown to other participants. Following the profiling questions, the main questionnaire includes five sections: Diagnosis, Treatment, Monitoring, Multidisciplinary care and Prognosis.

In each section, questions may ask you to respond using a single value (numeric response), by ranking options (ranking), selecting your level of agreement with a statement (Likert scale), or entering free text (open-ended questions). For further information, examples of these question types and the consensus definition for each question type please refer to Appendix 1.

This Delphi Panel is sponsored by Leadiant Biosciences
Date of Preparation: March 2019
GL-NP-1900002

First Round CTX Delphi Panel Questionnaire
Introduction continued

If you feel that you do not have sufficient expertise to answer an individual question, please select ‘Insufficient expertise’. Alternatively, if you do not wish to answer an individual question for any other reason, please select ‘Do not wish to answer’. If you would like to provide justification for your answers, or have any additional comments, please feel free to complete the available text boxes at the end of each section.

The responses and comments you provide throughout this questionnaire will be used to inform subsequent rounds of this Delphi panel.

Please note this first round questionnaire should take approximately 45 minutes to complete, and your responses will remain anonymous to the other Delphi panel participants. The questionnaire must be completed in a single sitting and responses will not be saved until you have finished the entire survey.

Adverse Event Reporting

Should you raise an adverse event/product complaint associated with the use of a Leadiant Biosciences medicinal product in a specific patient or group of patients, we will need to report this, even if it has already been reported by you directly to the company or the regulatory authorities using the MHRA's 'Yellow Card' system. If you decide to disclose your personal details in association with any adverse event/product complaint report, this information will be disclosed to the commissioning company. In such a situation you may be contacted specifically in relation to that adverse event. Everything else you contribute to the questionnaire will continue to remain confidential.

* 1. **Please tick the box to confirm that you agree for your details to be passed on to Leadiant Biosciences should an adverse event/product complaint associated with the use of a Leadiant Biosciences medicinal product be recorded**

☐ Yes

This Delphi Panel is sponsored by Leadiant Biosciences
Date of Preparation: March 2019
GL-NP-1900002

First Round CTX Delphi Panel Questionnaire
Profiling Questions

2. Please specify your role as a healthcare professional by selecting an option from the list:

If "Other", please specify below:

3. Please specify if you work at a CTX specialist centre/department or non-specialist (local) centre by selecting an option from the list:

If "Other", please specify below:

4. Please specify the country you practice in by selecting an option from the list:

If "Other", please specify below:

5. Please specify the approximate number of CTX patients you are currently treating and/or have treated in the past 10 years by selecting an option from the list:

6. Please specify whether you have cared for/treated adult (aged ≥18 years old) or paediatric patients (aged <18 years old), or both adult and paediatric patients, from the list below:

7. Please specify how many years you have been treating CTX patients for by selecting an option from the list:

This Delphi Panel is sponsored by Leadiant Biosciences
Date of Preparation: March 2019
GL-NP-1900002

First Round CTX Delphi Panel Questionnaire
Diagnosis

8. Please rank the following indicators in terms of their importance when considering a CTX diagnosis (*1=most important; 4=least important*).

- CYP27A1 genetic mutation
- An affected sibling
- Clinical signs and symptoms
- Biochemical pathogenesis

9. Please provide details of any additional important indicators to consider when diagnosing CTX in the text box below:

10. Please rank the following tests based on how often they are used to diagnose CTX in your experience (*1=most often, 4=least often*).

- Genetic testing
- Detection of urinary bile alcohols
- Biochemical testing – determination of serum cholestanol levels
- Conventional brain MRI

11. Please provide details of any additional tests used to diagnose CTX in the text box below:

This Delphi Panel is sponsored by Leadiant Biosciences
Date of Preparation: March 2019
GL-NP-1900002

First Round CTX Delphi Panel Questionnaire

Diagnosis

Please specify your level of agreement with the following statements by selecting an option from the dropdown list (*1=Strongly disagree; 6=Strongly agree*).

12. In the below table, please indicate symptoms that **paediatric** patients (aged <18 years old) present with, prior to a CTX diagnosis. *Please respond with a tick for each symptom type under the columns 1 – 6 (1=Strongly disagree; 6=Strongly agree)*.

	1- Strongly disagree	2- Disagree	3 - Somewhat disagree	4 - Somewhat agree	5 - Agree	6 - Strongly agree	Insufficient expertise	Do not wish to answer
Chronic diarrhoea	○	○	○	○	○	○	○	○
Bilateral juvenile cataracts	○	○	○	○	○	○	○	○
Early psychiatric symptoms (e.g. autism)	○	○	○	○	○	○	○	○
Mental retardation (e.g. learning difficulties)	○	○	○	○	○	○	○	○
Tendon xanthomas	○	○	○	○	○	○	○	○
Neonatal cholestatic jaundice	○	○	○	○	○	○	○	○

Please provide details of any additional symptoms that paediatric patients present with in the text box below:

This Delphi Panel is sponsored by Leadiant Biosciences
Date of Preparation: March 2019
GL-NP-1900002

First Round CTX Delphi Panel Questionnaire
Diagnosis

13. In the below table, please indicate symptoms that **adult** patients (aged ≥18 years old) present with, prior to a CTX diagnosis. *Please respond with a tick for each symptom type under the columns 1 – 6 (1=Strongly disagree; 6=Strongly agree)*.

	1 - Strongly disagree	2 - Disagree	3 - Somewhat disagree	4 - Somewhat agree	5 - Agree	6 - Strongly agree	Insufficient expertise	Do not wish to answer
Infantile-onset diarrhoea								
Childhood-onset cataracts								
Tendon xanthomas								
Early dementia								
Psychiatric symptoms								
Peripheral neuropathy								
Cerebellar signs								
Pyramidal signs								
Movement disorders (e.g. atypical parkinsonism)								
Epilepsy								

Please provide details of any additional symptoms that adult patients present with in the text box below:

14. All patients have elevated levels of serum cholestanol at the time of diagnosis. 4

This Delphi Panel is sponsored by Leadiant Biosciences
Date of Preparation: March 2019
GL-NP-1900002

First Round CTX Delphi Panel Questionnaire
Diagnosis

15. Brain MRI should be performed at the diagnosis stage as they can contribute to the diagnosis of CTX by revealing abnormally increased or decreased signals with characteristics distribution, but also to exclude other conditions.[5]

16. Measurement of serum cholestanol levels is the diagnostic maker of choice for CTX.[6]

17. Movement disorders can be considered as late CTX manifestations, however, CTX should be considered in the differential diagnosis of movement disorders, particularly in case of an early onset and when associated with other neurological features and/or with systemic features.[7]

18. DBS testing is the optimal method for screening of CTX in newborns. [6, 8]

Please provide details of any additional tests used to screen CTX in the text box below:

19. If you have any additional comments on the questions in this section, please feel free to add them to the following text box:

This Delphi Panel is sponsored by Leadiant Biosciences
Date of Preparation: March 2019
GL-NP-1900002

First Round CTX Delphi Panel Questionnaire
Treatment

20. Please rank the following factors in order of their impact on treatment outcomes in patients with CTX (*1=greatest impact; 3=least impact*).

- Age at diagnosis and treatment initiation
- Extent of neurological deterioration
- Cholestanol level at diagnosis

21. Please provide details of any additional factors that may have an impact on treatment outcomes in the text box below:

22. Please rank the following parameters in order of their utility for measuring treatment efficacy in patients with CTX (*1=most useful; 4=least useful*).

- Levels of serum cholestanol
- Levels of urinary bile alcohols
- Imaging (e.g. MRI) outcomes
- Clinical presentation/neurological examination

23. Please provide details of any additional parameters used to measure treatment efficacy in patients with CTX in the text box below:

This Delphi Panel is sponsored by Leadiant Biosciences
Date of Preparation: March 2019
GL-NP-1900002

First Round CTX Delphi Panel Questionnaire
Treatment

24. Please rank the following therapy options in order of their effectiveness for treating patients with CTX (*1=most effective; 5=least effective*).[9-11]

- CDCA alone
- CDCA and HMG-CoA reductase inhibitor
- Cholic acid alone
- Cholic acid and HMG-CoA reductase inhibitor
- LDL apheresis

25. Please provide details of any additional treatment options for patients with CTX in the text box below:

26. Please indicate when the most beneficial time to start CTX treatment is by ranking the below options (*1= greatest benefit; 4=least benefit*).

- From birth following a positive newborn screening test for CTX
- Upon CTX diagnosis (with or without symptom onset)
- Upon symptom onset in patients diagnosed with CTX
- Upon presentation of neurological symptoms in patients diagnosed with CTX

27. Please provide details of any additional scenarios when patients should start treatment:

This Delphi Panel is sponsored by Leadiant Biosciences
Date of Preparation: March 2019
GL-NP-1900002

First Round CTX Delphi Panel Questionnaire

Treatment

Please specify your level of agreement with the following statements by selecting an option from the dropdown list (*1=Strongly disagree; 6=Strongly agree*).

28. When, if at all, should CDCA treatment be discontinued? *Please enter text in the text box below.*

29. CDCA is a lifetime replacement therapy.[12]

30. The pathophysiological process in CTX patients may be reversed by CDCA, especially if treatment is initiated early in the disease process.[13]

31. Treatment with CDCA during pregnancy of mothers with CTX acts as an important means of protection against damage to the fetus and miscarriage.[14]

If you have any additional comments on the questions in this section, please feel free to add them to the following text box:

This Delphi Panel is sponsored by Leadiant Biosciences
Date of Preparation: March 2019
GL-NP-1900002

First Round CTX Delphi Panel Questionnaire

Monitoring

In this section, please consider adult and paediatric patients diagnosed with CTX who are currently receiving CDCA, unless otherwise specified.

32. Please indicate the number of times per year that **paediatric** patients (aged <18 years old) should be monitored for the types of symptoms described below. *Please respond with a single value.*

Central and peripheral nervous system

Ocular system

Cardiovascular system

Skeletal system

Pulmonary system

Enterohepatic system

33. Please tick **one** of the options below if you did not respond to the previous question:

☐ Insufficient expertise

☐ Do not wish to answer

Please provide the frequency (per year) of any additional symptom types that paediatric patients with CTX should be monitored for, in the text box below:

This Delphi Panel is sponsored by Leadiant Biosciences
Date of Preparation: March 2019
GL-NP-1900002

First Round CTX Delphi Panel Questionnaire
Monitoring

34. Please indicate the number of times per year that **adult** patients (aged ≥18 years old) should be monitored for the types of symptoms described below. *Please respond with a single value.*

Central and peripheral nervous system

Ocular system

Cardiovascular system

Skeletal system

Pulmonary system

Enterohepatic system

35. Please tick **one** of the options below if you did not respond to the previous question:

- [ ] Insufficient expertise
- [ ] Do not wish to answer

Please provide the frequency (per year) of any additional symptom types that adult patients with CTX should be monitored for, in the text box below:

This Delphi Panel is sponsored by Leadiant Biosciences
Date of Preparation: March 2019
GL-NP-1900002

First Round CTX Delphi Panel Questionnaire
Monitoring

36. Please rank the following examinations and tests in terms of their importance for monitoring patients with CTX (*1=most important; 5=least important*).

- Cholestanol plasma concentration
- Brain MRI
- Neurologic (and if necessary neuropsychologic evaluation)
- Liver function tests (LFTs)
- Urinary bile alcohol concentration

This Delphi Panel is sponsored by Leadiant Biosciences
Date of Preparation: March 2019
GL-NP-1900002

First Round CTX Delphi Panel Questionnaire
Monitoring

37. Please indicate the number of times per year that **paediatric** patients (aged <18 years old) should undergo the types of tests/examinations described below. *Please respond with a single value.*

Cholestanol plasma concentration

Urinary bile alcohol concentration

Brain MRI

Neurologic (and if necessary neuropsychologic evaluation)

Liver function tests (LFTs)

38. Please tick **one** of the options below if you did not respond to the previous question:

☐ Insufficient expertise

☐ Do not wish to answer

Please provide the frequency (per year) of any additional tests/examinations that paediatric patients with CTX should receive, in the text box below:

This Delphi Panel is sponsored by Leadiant Biosciences
Date of Preparation: March 2019
GL-NP-1900002

First Round CTX Delphi Panel Questionnaire
Monitoring

39. Please indicate the number of times per year that **adult** patients (aged ≥18 years old) should undergo the types of tests/examinations described below. *Please respond with a single value.*

Cholestanol plasma concentration

Urinary bile alcohol concentration

Brain MRI

Neurologic (and if necessary neuropsychologic evaluation)

Liver function tests (LFTs)

40. Please tick **one** of the options below if you did not respond to the previous question:

- [ ] Insufficient expertise
- [ ] Do not wish to answer

Please provide the frequency (per year) of any additional tests/examinations that adult patients with CTX should receive, in the text box below:

This Delphi Panel is sponsored by Leadiant Biosciences
Date of Preparation: March 2019
GL-NP-1900002

First Round CTX Delphi Panel Questionnaire

Monitoring

Please specify your level of agreement with the following statements by selecting an option from the dropdown list (*1=Strongly disagree; 6=Strongly agree*).

41. Transcranial magnetic stimulation (TMS) is a useful tool for evaluating improvements in pyramidal function in patients receiving CDCA.[15]

42. Treatment adherence can be improved by providing CTX patients with support and intensive education.[14]

43. T_2-weighted MRIs allow tracking of CTX disease progression with a greater sensitivity than clinical scales, therefore, should be used during follow-up of CTX patients, as well as normal MRIs.[5,16]

44. Pre-marital genetic counselling should be recommended to high-risk populations e.g. patients of Israeli or Moroccan origin.[14]

If you have any additional comments on the questions in this section, please feel free to add them to the following text box:

This Delphi Panel is sponsored by Leadiant Biosciences
Date of Preparation: March 2019
GL-NP-1900002

First Round CTX Delphi Panel Questionnaire
Multidisciplinary care

45. Please rank the importance of the following health care professionals in the **diagnosis** of CTX in **paediatric** patients (aged <18 years old) (*1=most important; 5=least important*).

- Neurologist
- Neuroradiologist
- Paediatrician (e.g. paediatric metabolic specialist or paediatric gastroenterologist)
- Geneticist
- Ophthalmologist

46. Please provide details of any additional important health care professionals involved in the **diagnosis** of CTX in **paediatric** patients (aged <18 years old) in the text box below:

First Round CTX Delphi Panel Questionnaire
Multidisciplinary care

47. Please rank the importance of the following health care professionals in the **diagnosis** of CTX in **adult** patients (aged ≥18 years old) (*1=most important; 7=least important*).

- Neurologist
- Neuroradiologist
- Geneticist
- Ophthalmologist
- Metabolic specialist
- Gastroenterologist
- Orthopaedic surgeon

48. Please provide details of any additional important health care professionals involved in the **diagnosis** of CTX in **adult** patients (aged ≥18 years old) in the text box below:

This Delphi Panel is sponsored by Leadiant Biosciences
Date of Preparation: March 2019
GL-NP-1900002

First Round CTX Delphi Panel Questionnaire
Multidisciplinary care

49. Please rank the importance of the following health care professionals in the **treatment** of CTX in **paediatric** patients (aged <18 years old) (*1=most important; 3=least important*).

- Neurologist
- Neuroradiologist
- Paediatrician (e.g. paediatric metabolic specialist or paediatric gastroenterologist)

50. Please provide details of any additional important health care professionals involved in the **treatment** of CTX in **paediatric** patients (aged <18 years old) in the text box below:

51. Please rank the importance of the following health care professionals in the **treatment** of CTX in **adult** patients (aged ≥18 years old) (*1=most important; 4=least important*).

- Neurologist
- Neuroradiologist
- Metabolic specialist
- Gastroenterologist

52. Please provide details of any additional important health care professionals involved in the **treatment** of CTX in **adult** patients (aged ≥18 years old) in the text box below:

This Delphi Panel is sponsored by Leadiant Biosciences
Date of Preparation: March 2019
GL-NP-1900002

First Round CTX Delphi Panel Questionnaire
Multidisciplinary care

53. Please rank the importance of the following health care professionals in the **follow-up** of CTX in **paediatric** patients (aged <18 years old) (*1=most important; 4=least important*).

- Neurologist
- Neuroradiologist
- Paediatrician (e.g. paediatric metabolic specialist or paediatric gastroenterologist)
- Ophthalmologist

54. Please provide details of any additional important health care professionals involved in the **follow-up** of CTX in **paediatric** patients (aged <18 years old) in the text box below:

This Delphi Panel is sponsored by Leadiant Biosciences
Date of Preparation: March 2019
GL-NP-1900002

First Round CTX Delphi Panel Questionnaire

Multidisciplinary care

55. Please rank the importance of the following health care professionals in the **follow-up** of CTX in **adult** patients (aged ≥18 years old) (*1=most important; 6=least important*).

- Neurologist
- Neuroradiologist
- Ophthalmologist
- Cardiologist
- Gastroenterologist
- Metabolic specialist

56. Please provide details of any additional important health care professionals involved in the **follow-up** of CTX in **adult** patients (aged ≥18 years old) in the text box below:

This Delphi Panel is sponsored by Leadiant Biosciences
Date of Preparation: March 2019
GL-NP-1900002

First Round CTX Delphi Panel Questionnaire
Multidisciplinary care

57. Please indicate the number of visits per year that **paediatric** patients (aged <18 years old) should attend at a specialist CTX centre/department and a local centre, fo follow-up care. *Please respond with a single value.*

Specialist CTX centre/department

Local centre

58. Please tick **one** of the options below if you did not respond to the previous question:

- [ ] Insufficient expertise
- [ ] Do not wish to answer

This Delphi Panel is sponsored by Leadiant Biosciences
Date of Preparation: March 2019
GL-NP-1900002

First Round CTX Delphi Panel Questionnaire
Multidisciplinary care

59. Please indicate the number of visits per year that **adult** patients (aged ≥18 years old) should attend at a specialist CTX centre/department and a local centre, for follow-up care. *Please respond with a single value.*

Specialist CTX centre/department

Local centre

60. Please tick **one** of the options below if you did not respond to the previous question:

☐ Insufficient expertise

☐ Do not wish to answer

61. If you have any additional comments on the questions in this section, please feel free to add them to the following text box:

This Delphi Panel is sponsored by Leadiant Biosciences
Date of Preparation: March 2019
GL-NP-1900002

First Round CTX Delphi Panel Questionnaire

Prognosis

Please specify your level of agreement with the following statements by selecting an option from the dropdown list (*1=Strongly disagree; 6=Strongly agree*).

62. Please indicate which of the below therapy options improves/stabilises prognosis in the majority of CTX patients.[17-19]

	1 - Strongly disagree	2 - Disagree	3 - Somewhat disagree	4 - Somewhat agree	5 - Agree	6 - Strongly agree	Insufficient expertise	Do not wish to answer
CDCA alone	○	○	○	○	○	○	○	○
CDCA and HMG-CoA reductase inhibitor	○	○	○	○	○	○	○	○
Cholic acid alone	○	○	○	○	○	○	○	○
Cholic acid and HMG-CoA reductase inhibitor	○	○	○	○	○	○	○	○
LDL apheresis	○	○	○	○	○	○	○	○

63. Reducing plasma cholestanol concentrations slows down the progression of CTX.

64. A correlation between the progression of clinical and neuroradiological symptoms exists in CTX patients.

65. CTX patients who start treatment after significant neurological pathology is established, have a worse prognosis compared to patients who started treatment as early as possible.

This Delphi Panel is sponsored by Leadiant Biosciences
Date of Preparation: March 2019
GL-NP-1900002

First Round CTX Delphi Panel Questionnaire
Prognosis

66. The neurological stability of patients with CTX is determined by brain MRI.

67. In CTX patients, the absence of dentate nuclei signal alteration in brain MRI is an indicator of better prognosis.[20]

68. During follow-up brain MRI examinations, increased atrophy and/or signal alterations are present in CTX patients who have deteriorating neurological symptoms.

69. CTX patients showing MRI evidence of cerebellar vacuolation should be monitored more strictly over time as it is considered a prognostic marker.[13, 20]

70. If you have any additional comments on the questions in this section, please feel free to add them to the following text box:

This Delphi Panel is sponsored by Leadiant Biosciences
Date of Preparation: March 2019
GL-NP-1900002

First Round CTX Delphi Panel Questionnaire
References

1. Resemann H, Clements S, Griffiths A, et al. Reporting of Delphi Methods to Achieve Consensus on Guidelines in Rare Diseases. Presented at the 2018 European Meeting of the International Society for Medical Publication Professionals (ISMPP), London, UK. 2018.

2. McMillan SS, King M, Tully MP. How to use the nominal group and Delphi techniques. International Journal of Clinical Pharmacy 2016;38:655-662.

3. Hsu C, Sandford B. The Delphi Technique: Making Sense of Consensus. PARE 2007;12:1-8.

4. Mignarri A, Gallus GN, Dotti MT, et al. A suspicion index for early diagnosis and treatment of cerebrotendinous xanthomatosis. J Inherit Metab Dis 2014;37:421-9.

5. Mascalchi M, Vella A. Neuroimaging Applications in Chronic Ataxias, 2018.

6. Bleyle L, Huidekoper HH, Vaz FM, et al. Update on newborn dried bloodspot testing for cerebrotendinous xanthomatosis: An available high-throughput liquid-chromatography tandem mass spectrometry method. Molecular Genetics and Metabolism Reports 2016;7:11-15.

7. Stelten BML, van de Warrenburg BPC, Wevers RA, et al. Movement disorders in cerebrotendinous xanthomatosis. Parkinsonism & Related Disorders 2018.

8. DeBarber AE, Kalfon L, Fedida A, et al. Newborn screening for cerebrotendinous xanthomatosis is the solution for early identification and treatment. J Lipid Res 2018;59:2214-2222.

9. Verrips A, Wevers RA, Van Engelen BG, et al. Effect of simvastatin in addition to chenodeoxycholic acid in patients with cerebrotendinous xanthomatosis. Metabolism 1999;48:233-8.

10. Sekijima Y, Koyama S, Yoshinaga T, et al. Nationwide survey on cerebrotendinous xanthomatosis in Japan. J Hum Genet 2018;63:271-280.

11. Koopman BJ, Wolthers BG, van der Molen JC, et al. Bile acid therapies applied to patients suffering from cerebrotendinous xanthomatosis. Clinica Chimica Acta 1985;152:115-122.

12. Rosenberg R, Pascual J. Rosenberg's molecular and genetic basis of neurological and psychiatric disease. Elsevier 2015;Fifth edition:596-597.

13. Amador MdM, Masingue M, Debs R, et al. Treatment with chenodeoxycholic acid in cerebrotendinous xanthomatosis: clinical, neurophysiological, and quantitative brain structural outcomes. Journal of Inherited Metabolic Disease 2018;41:799-807.

14. Yahalom G, Tsabari R, Molshatzki N, et al. Neurological outcome in cerebrotendinous xanthomatosis treated with chenodeoxycholic acid: early versus late diagnosis. Clinical neuropharmacology 2013;36:78-83.

This Delphi Panel is sponsored by Leadiant Biosciences
Date of Preparation: March 2019
GL-NP-1900002

First Round CTX Delphi Panel Questionnaire
References

15. Nie S, Chen G, Cao X, et al. Cerebrotendinous xanthomatosis: a comprehensive review of pathogenesis, clinical manifestations, diagnosis, and management. Orphanet journal of rare diseases 2014;9:179.

16. Pilo-de-la-Fuente B, Jimenez-Escrig A, Lorenzo J, et al. Cerebrotendinous xanthomatosis in Spain: clinical, prognostic, and genetic survey. European journal of neurology 2011;18:1203-1211.

17. Mignarri A, Rossi S, Ballerini M, et al. Clinical relevance and neurophysiological correlates of spasticity in cerebrotendinous xanthomatosis. Journal of neurology 2011;258:783-790.

18. Mimura Y, Kuriyama M, Tokimura Y, et al. Treatment of cerebrotendinous xanthomatosis with low-density lipoprotein (LDL)-apheresis. J Neurol Sci 1993;114:227-30.

19. Duell PB, Salen G, Eichler FS, et al. Diagnosis, treatment, and clinical outcomes in 43 cases with cerebrotendinous xanthomatosis. Journal of Clinical Lipidology 2018.

20. Mignarri A, Dotti MT, Federico A, et al. The spectrum of magnetic resonance findings in cerebrotendinous xanthomatosis: redefinition and evidence of new markers of disease progression. Journal of neurology 2017;264:862-874.

This Delphi Panel is sponsored by Leadiant Biosciences
Date of Preparation: March 2019
GL-NP-1900002

First Round CTX Delphi Panel Questionnaire
Appendix 1

This questionnaire uses four different question types to gain consensus:

- **Question Type 1: Numeric response**
- You are asked to respond with a single value e.g. “Please indicate the number of times per year that adult and paediatric patients should be monitored for the types of symptoms described in column 1”
- Consensus definition: After the first round, when all responses have been received, a range of numbers will be specified within which ≥70% of participants’ responses must be included in order to have achieved consensus
- **Question Type 2: Ranking**
- You are asked to rank the options e.g. “Please rank these four examinations in order of importance”
- Consensus definition: Kendall’s W stat ≥0.7
- This is a measure from 0 to 1. If the W stat is 1, everyone has the same ordering of preferences
- **Question Type 3: Likert scale**
- You are asked to select your level of agreement with a statement, e.g. “Please specify your level of agreement with the following statements by selecting an option from the dropdown list (1 - Strongly disagree; 6 - Strongly agree)”
- Consensus definition: ≥70% of participants choose the same option
- If ≥70% of participants respond with ‘1 - Strongly disagree’ or ‘2 - Disagree’, the consensus is disagreement with the specified statement
- If <70% of participants respond with ‘5 - Agree’ or ‘6 - Strongly agree’, the consensus is agreement with the specified statement
- If <70% of participant responses agree with each other, there is no consensus with the specified statement
- Please see likert scale on the next page

This Delphi Panel is sponsored by Leadiant Biosciences
Date of Preparation: March 2019
GL-NP-1900002

First Round CTX Delphi Panel Questionnaire
Appendix 1

71. Question Type 3: Likert scale

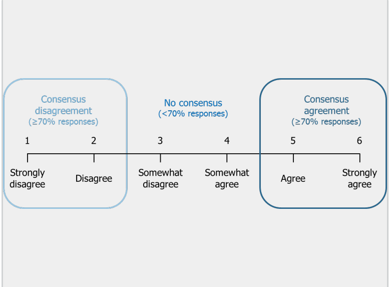

Likert scale

- **Question Type 4: Open-ended questions**
- You are asked to provide a free text answer (i.e. there are no options to select from and the participant can add any response in as much detail as they wish)
- There is no consensus definition for open-ended questions. These questions are used to gather ideas from participants so more specific questions can be generated for the second round questionnaire

Please also note the following:

- Any question that a respondent answers with “Insufficient expertise” will not be included in the statistical analysis (applicable for all question types 1–3)
- Any question that a respondent answers with “Do not wish to answer” will be considered ‘neutral’ responses (i.e. the respondent neither agrees nor disagrees) (applicable to question type 3 only)

This Delphi Panel is sponsored by Leadiant Biosciences
Date of Preparation: March 2019
GL-NP-1900002

First Round CTX Delphi Panel Questionnaire
Appendix 2

1. Batta A, Salen G, Tint GS. Hydrophilic 7β-Hydroxy Bile Acids, Lovastatin, and Cholestyramine Are Ineffective in the Treatment of Cerebrotendinous Xanthomatosis. Metabolism: Clinical and Experimental. 2004;53(5):556-562.

2. Beppu T, Seyama Y, Kasama T. Serum bile acid profiles in cerebrotendinous xanthomatosis. Clinica Chimica Acta. 1982;118(2-3):167-175.

3. Berginer VM, Radwan H, Korczyn AD. EEG in cerebrotendinous xanthomatosis (CTX). Clinical EEG Electroencephalography. 1982;13(2):89-96.

4. Berginer VM, Salen G, Shefer S. Long-term treatment of cerebrotendinous xanthomatosis with chenodeoxycholic acid. New England Journal of Medicine. 1984;311(26):1649-1652.

5. Bleyle L, Huidekoper HH, Vaz FM, Singh R, Steiner RD, Debarber AE. Update on newborn dried bloodspot testing for cerebrotendinous xanthomatosis: An available high-throughput liquid-chromatography tandem mass spectrometry method. Molecular Genetics and Metabolism Reports. 2016;7:11-15.

6. DeBarber AE, Kalfon L, Fedida A, Sheffer VF, Haroush SB, Chasnyk N, Shuster E, Mandel H, Jeffries K, Shinwell ES, Falik-Zaccai TC. Newborn screening for cerebrotendinous xanthomatosis is the solution for early identification and treatment. Journal of Lipid Research. 2018;59(11):2214-2222.

7. DeBarber AE, Luo J, Star-Weinstock M, Purkayastha S, Geraghty MT, Chiang J, Merkens LS, Pappu AS, Steiner RD. A blood test for cerebrotendinous xanthomatosis with potential for disease detection in newborns. Journal of Lipid Research. 2014;55(1):146-154.

8. Del Mar Amador M, Masingue M, Debs R, Lamari F, Perlbarg V, Roze E, Degos B, Mochel F. Treatment with chenodeoxycholic acid in cerebrotendinous xanthomatosis: clinical, neurophysiological, and quantitative brain structural outcomes. Journal of Inherited Metabolic Disease. 2018;41(5):799-807

9. Duell PB, Salen G, Eichler FS, DeBarber AE, Connor SL, Casaday L, Jayadev S, Kisanuki Y, Lekprasert P, Malloy MJ, Ramdhani RA, Ziajka PE, Quinn JF, Su KG, Geller AS, Diffenderfer R, Schaefer EJ. Diagnosis, treatment, and clinical outcomes in 43 cases with cerebrotendinous xanthomatosis. Journal of Clinical Lipidology. 2018;12(5):1169-1178.

10. Ginanneschi F, Mignarri A, Mondelli M, Gallus GN, Del Puppo M, Giorgi S, Federico A, Rossi A, Dotti MT. Polyneuropathy in cerebrotendinous xanthomatosis and response to treatment with chenodeoxycholic acid. Journal of Neurology. 2013;260(1):268-274.

This Delphi Panel is sponsored by Leadiant Biosciences
Date of Preparation: March 2019
GL-NP-1900002

11. Inglese M, De Stefano N, Pagani E, Dotti MT, Comi G, Federico A, Filippi M. Quantification of brain damage in cerebrotendinous xanthomatosis with magnetization transfer MR imaging. American Journal of Neuroradiology. 2003;24(3):495-500.

12. Kohler W, Curiel J, Vanderver A. Adulthood leukodystrophies. Nature Reviews. 2018;14(2):94-105.

13. Maschalchi M, Vella A. Neuroimaging Applications in Chronic Ataxias. International Review of Neurobiology. 2018;143:109-162.

14. Mignarri A, Dotti MT, Federico A, De Stefano N, Battaglini M, Grazzini I, Galluzzi P, Monti L. The spectrum of magnetic resonance findings in cerebrotendinous xanthomatosis: redefinition and evidence of new markers of disease progression. Journal of Neurology. 2017;264(5):862-874.

15. Mignarri A, Gallus GN, Dotti MT, Federico A. A suspicion index for early diagnosis and treatment of cerebrotendinous xanthomatosis. Journal of Inherited Metabolic Disease. 2014;37(3):421-429.

16. Mignarri A, Magni A, Del Puppo M, Gallus GN, Bjorkhem I, Federico A, Dotti MT. Evaluation of cholesterol metabolism in cerebrotendinous xanthomatosis. Journal of Inherited Metabolic Disease. 2016;39(1):75-83.

17. Mignarri A, Rossi S, Ballerini M, Gallus GN, Del Puppo M, Galluzzi P, Federico A, Dotti MT. Clinical relevance and neurophysiological correlates of spasticity in cerebrotendinous xanthomatosis. Journal of Neurology. 2011;258(5):783-790.

18. Nei S, Chen G, Cao X, Zhang Y. Cerebrotendinous xanthomatosis: a comprehensive review of pathogenesis, clinical manifestations, diagnosis, and management. Orphanet journal of rare diseases. 2014;9:179.

19. Pilo-de-la-Fuente B, Jimenez-Escrig A, Lorenzo JR, Pardo J, Arias M, Ares-Luque A, Duarte J, Muniz-Perez S, Sobrido MJ. Cerebrotendinous xanthomatosis in Spain: Clinical, prognostic, and genetic survey. European Journal of Neurology. 2011;18(10):1203-1211.

20. Pitt JJ. High-throughput urine screening for Smith-Lemli-Opitz syndrome and cerebrotendinous xanthomatosis using negative electrospray tandem mass spectrometry. Clinica Chimica Acta. 2007;380(1-2):81-88.

This Delphi Panel is sponsored by Leadiant Biosciences
Date of Preparation: March 2019
GL-NP-1900002

21. Salen G, Shefer S, Berginer V. Biochemical abnormalities in cerebrotendinous xanthomatosis. Developmental Neuroscience. 1991;13(4-5):363-370.

22. Salen G, Steiner RD. Epidemiology, diagnosis, and treatment of cerebrotendinous xanthomatosis (CTX). Journal of Inherited Metabolic Disease. 2017;40(6):771-781.

23. Stelten BML, Bonnot O, Huidekoper HH, van Spronsen FJ, van Hasselt PM, Kluijtmans LAJ, Wevers RA, Verrips A. Autism spectrum disorder: an early and frequent feature in cerebrotendinous xanthomatosis. Journal of Inherited Metabolic Disease. 2018;41(4):641-646.

24. Stelten BML, van de Warrenburg BPC, Wevers RA, Verrips A. Movement disorders in cerebrotendinous xanthomatosis. Parkinsonism and Related Disorders.2018:1-5.

25. Vaz FM, Bootsma AH, Kulik W, Verrips A, Wevers RA, Schielen PC, DeBarber AE, Huidekoper HH. A newborn screening method for cerebrotendinous xanthomatosis using bile alcohol glucuronides and metabolite ratios. Journal of Lipid Research. 2017;58(5):1002-1007.

26. Verrapis A, Van Engelen BGM, Wevers RA, Van Geel BM, Cruysberg JRM, Van Den Heuvel LPWJ, Keyser A, Gabreels FJM. Presence of diarrhea and absence of tendon xanthomas in patients with cerebrotendinous xanthomatosis. Archives of Neurology. 2000;57(4):520-524.

27. Verrapis A, Wevers RA, Van Engelen BGM, Keyser A, Wolthers BG, Barkhof F, Stalenhoef A, De Graaf R, Janssen-Zijlstra F, Van Spreeken A, Gabreels FJM. Effect of simvastatin in addition to chenodeoxycholic acid in patients with cerebrotendinous xanthomatosis. Metabolism: Clinical and Experimental. 1999;48(2):233-238.

28. Waterreus RJ, Koopman BJ, Wolthers BG, Oosterhuis HJGH. Cerebrotendinous xanthomatosis (CTX): A clinical survey of the patient population in the Netherlands. Clinical Neurology and Neurosurgery. 1987;89(3):169-175.

29. Yahalom G, Tsabari R, Molshatzki N, Ephraty L, Cohen H, Hassin-Baer S. Neurological outcome in cerebrotendinous xanthomatosis treated with chenodeoxycholic acid: Early versus late diagnosis. Clinical Neuropharmacology. 2013;36(3):78-83.

30. Zubarioglu T, Kiykim E, Yesil G, Demircioglu D, Cansever MS, Yalcinkaya C, Aktuglu-Zeybek C. Early diagnosed cerebrotendinous xanthomatosis patients: clinical, neuroradiological characteristics and therapy results of a single center from Turkey. Acta Neurologica Belgica. 2017:1-8.

This Delphi Panel is sponsored by Leadiant Biosciences
Date of Preparation: March 2019
GL-NP-1900002

Round 2 Questionnaire

Introduction

Thank you for completing Round 1 of the Delphi panel and for taking the time to complete this Round 2 questionnaire. The aim of the Delphi panel is to achieve consensus on best practices for diagnosing, treating and managing patients with cerebrotendinous xanthomatosis (CTX).

Round 2 Questionnaire Development

Questions that achieved consensus in Round 1 have not been included in Round 2. Questions that did not achieve consensus in Round 1 have been asked again in this Round 2 questionnaire. Based on Round 1 responses and free-text comments, these questions have been restated, rephrased, formulated into a new question type or split into multiple related questions. The questions in Round 2 have been validated by a clinical expert in CTX.

Questionnaire Structure and Format

The Round 2 questionnaire includes five sections: Diagnosis, Treatment, Monitoring, Multidisciplinary care and Prognosis. In each section, questions may ask you to respond by ranking options (ranking), selecting a percentage category (proportion) or selecting your level of agreement with a statement (Likert scale). For further details of how the consensus threshold will be applied to the three different question types please see the Appendix.

If you feel that you do not have sufficient expertise to answer an individual question, please select 'Insufficient expertise'. Alternatively, if you do not wish to answer an individual question for any other reason, please select 'Do not wish to answer'. If you would like to provide justification for your answers, or have any additional comments, please feel free to complete the available text boxes at the end of each question or section.

The responses and comments you provide throughout this questionnaire will be used to inform the third and final round of this Delphi panel.

Completing the Questionnaire

Please provide responses to all questions based on what you **believe to be best practice**.

The following definitions apply throughout the questionnaire:

1. Adult patients with CTX: aged ≥18 years
2. Paediatric patients with CTX: aged <18 years

In line with Delphi methodology, all participants should complete this Round 2 questionnaire taking into account the group results from the previous round, to provide you with an opportunity to reassess your initial judgements. In this Round 2 questionnaire we refer to slides from the Round 1 Results Summary slideset, which is attached to your Round 2 invitation email. Slide numbers are shown at the end of each question in square brackets, e.g. [Slide 10]. Therefore, we **recommend that you review this slideset while completing the Round 2 questionnaire**.

Please note, a small number of participants only provided partial responses for ranking questions (i.e. not all options were ranked) in Round 1. Therefore, ranking questions were not used to determine consensus in Round 1. These questions have been restated or rephrased in Round 2, based on the responses received in Round 1. **Please provide responses to all questions and options listed**.

Please note this Round 2 questionnaire should take approximately 20–25 minutes to complete, and your responses will remain anonymous to the other Delphi panel participants. The questionnaire must be completed in a single sitting and responses will not be saved until you have finished the entire survey.

This Delphi Panel is sponsored by Leadiant Biosciences
Date of preparation: July 2019
GL-NP-1900011

Adverse Event Reporting

Should you raise an adverse event/product complaint associated with the use of a Leadiant Biosciences medicinal product in a specific patient or group of patients, we will need to report this, even if it has already been reported by you directly to the company or the regulatory authorities using the MHRA's 'Yellow Card' system. If you decide to disclose your personal details in association with any adverse event/product complaint report, this information will be disclosed to the commissioning company. In such a situation you may be contacted specifically in relation to that adverse event. Everything else you contribute to the questionnaire will continue to remain confidential.

* 1. **Please tick the box to confirm that you agree for your details to be passed on to Leadiant Biosciences should an adverse event/product complaint associated with the use of a Leadiant Biosciences medicinal product be recorded.**

☐ Yes

This Delphi Panel is sponsored by Leadiant Biosciences
Date of preparation: July 2019
GL-NP-1900011

Diagnosis

* 2. *When answering this question, consider a scenario where a patient has been referred to you following the onset of symptoms.*

Please rank the following **indicators** in order of which has the greatest diagnostic value, when considering a CTX diagnosis (*1=greatest diagnostic value; 5=least diagnostic value*). [Slide 12]

	1 – Greatest diagnostic value	2	3	4	5 – Least diagnostic value	Insufficient expertise	Do not wish to answer
CYP27A1 genetic mutation	○	○	○	○	○	○	○
An affected sibling	○	○	○	○	○	○	○
Clinical signs and symptoms	○	○	○	○	○	○	○
Biochemical pathogenesis (assessed through biochemical testing)	○	○	○	○	○	○	○
Brain MRI findings	○	○	○	○	○	○	○

Please feel free to provide any additional comments on this question in the text box below:

* 3. Please rank the following **tests/examinations** in order of importance when confirming a CTX diagnosis (*1=most important; 5=least important*). [Slide 13]

	1 – Most important	2	3	4	5 – Least important	Insufficient expertise	Do not wish to answer
Genetic testing alone	◯	◯	◯	◯	◯	◯	◯
Determination of serum cholestanol levels	◯	◯	◯	◯	◯	◯	◯
Detection of urinary bile alcohols	◯	◯	◯	◯	◯	◯	◯
Determination of plasma bile acids (mainly cholic acid and chenodeoxycholic acid)	◯	◯	◯	◯	◯	◯	◯
Conventional brain MRI	◯	◯	◯	◯	◯	◯	◯

Please feel free to provide any additional comments on this question in the text box below:

* 4. Please indicate the proportion of **paediatric** patients that present with the following symptoms, prior to a CTX diagnosis (*Please respond with a tick for each symptom type*). [Slide 14]

	0–24%	25–49%	50–74%	75–100%	Insufficient expertise	Do not wish to answer
Early psychiatric symptoms (e.g. autism)	◯	◯	◯	◯	◯	◯
Tendon xanthomas	◯	◯	◯	◯	◯	◯
Neonatal cholestatic jaundice	◯	◯	◯	◯	◯	◯
Cerebellar system findings (e.g. ataxia symptoms and tremor)	◯	◯	◯	◯	◯	◯
Epilepsy	◯	◯	◯	◯	◯	◯
Peripheral neuropathy where Z-scores are below the expected range for age in bone mineral density (BMD)	◯	◯	◯	◯	◯	◯

Please feel free to provide any additional comments on this question in the text box below:

* 5. Please indicate the proportion of **adult** patients that present with the following symptoms, prior to a CTX diagnosis (*Please respond with a tick for each symptom type*). [Slide 15]

	0–24%	25–49%	50–74%	75–100%	Insufficient expertise	Do not wish to answer
Early-onset dementia	◯	◯	◯	◯	◯	◯
Early-onset movement disorder (e.g. atypical parkinsonism)	◯	◯	◯	◯	◯	◯
Epilepsy	◯	◯	◯	◯	◯	◯

Please feel free to provide any additional comments on this question in the text box below:

6. If you have any additional comments on the questions in this section, please feel free to add them to the following text box:

This Delphi Panel is sponsored by Leadiant Biosciences
Date of preparation: July 2019
GL-NP-1900011

Treatment

* 7. Please rank the following factors in order of their impact on treatment outcomes in patients with CTX (*1=greatest impact; 5=least impact*). [Slide 17]

	1 – Greatest impact	2	3	4	5 – Least impact	Insufficient expertise	Do not wish to answer
Age at diagnosis and treatment initiation	○	○	○	○	○	○	○
Extent of neurological deterioration	○	○	○	○	○	○	○
Cholestanol level at diagnosis	○	○	○	○	○	○	○
Treatment compliance	○	○	○	○	○	○	○
Characteristics of cerebellar signal abnormalities	○	○	○	○	○	○	○

Please feel free to provide any additional comments on this question in the text box below:

* 8. Please rank the following parameters in order of their usefulness for measuring treatment efficacy in patients with CTX (*1=most useful; 5=least useful*). [Slide 18]

	1 – Most useful	2	3	4	5 – Least useful	Insufficient expertise	Do not wish to answer
Levels of serum cholestanol alone	○	○	○	○	○	○	○
Clinical presentation/neurological examination	○	○	○	○	○	○	○
Brain MRI	○	○	○	○	○	○	○
Levels of urinary bile alcohols	○	○	○	○	○	○	○
Electrophysiological examinations (e.g. electromyography, nerve conduction velocity, electroencephalography)	○	○	○	○	○	○	○

Please feel free to provide any additional comments on this question in the text box below:

* 9. Please rank the following therapy options in order of their effectiveness for treating the underlying biochemical abnormalities in CTX (*1=most effective; 5=least effective*).[1-3] [Slide 19]

	1 – Most effective	2	3	4	5 – Least effective	Insufficient expertise	Do not wish to answer
CDCA alone	◯	◯	◯	◯	◯	◯	◯
CDCA and HMG-CoA reductase inhibitor	◯	◯	◯	◯	◯	◯	◯
Cholic acid alone	◯	◯	◯	◯	◯	◯	◯
Cholic acid and HMG-CoA reductase inhibitor	◯	◯	◯	◯	◯	◯	◯
LDL apheresis	◯	◯	◯	◯	◯	◯	◯

Please feel free to provide any additional comments on this question in the text box below:

* 10. Please indicate when the most beneficial time to start CTX treatment is by ranking the below options (*1=most beneficial; 4=least beneficial*). [Slide 20]

	1 – Most beneficial	2	3	4 – Least beneficial	Insufficient expertise	Do not wish to answer
From birth following a positive newborn screening test for CTX	◯	◯	◯	◯	◯	◯
Upon CTX diagnosis (with or without symptom onset)	◯	◯	◯	◯	◯	◯
Upon symptom onset in patients diagnosed with CTX	◯	◯	◯	◯	◯	◯
Upon presentation of neurological symptoms in patients diagnosed with CTX	◯	◯	◯	◯	◯	◯

Please feel free to provide any additional comments on this question in the text box below:

* 11. Research indicates that treating CTX mothers with CDCA during pregnancy acts as an important means of protection against damage to the fetus and miscarriage.[4] *Please specify your level of agreement by selecting an option from the dropdown list (1 – Strongly disagree; 6 – Strongly agree)*. [Slide 22]

Please feel free to provide any additional comments on this question in the text box below:

12. If you have any additional comments on the questions in this section, please feel free to add them to the following text box:

This Delphi Panel is sponsored by Leadiant Biosciences
Date of preparation: July 2019
GL-NP-1900011

Monitoring

In this section, please consider adult and paediatric patients diagnosed with CTX who are currently receiving CDCA, unless otherwise specified.

* 13. During the early stages of treatment, **paediatric** patients should be monitored for the types of symptoms listed below **1–2 times per year**. *Please respond with a tick for each symptom type* (*1 – Strongly disagree; 6 – Strongly agree*). [Slide 24]

	1 – Strongly disagree	2 – Disagree	3 – Somewhat disagree	4 – Somewhat agree	5 – Agree	6 – Strongly agree	Insufficient expertise	Do not wish to answer
Central and peripheral nervous system	○	○	○	○	○	○	○	○
Ocular system	○	○	○	○	○	○	○	○
Cardiovascular system	○	○	○	○	○	○	○	○
Skeletal system	○	○	○	○	○	○	○	○
Pulmonary system	○	○	○	○	○	○	○	○
Enterohepatic system	○	○	○	○	○	○	○	○
Cognitive performance (e.g. learning difficulties)	○	○	○	○	○	○	○	○

Please feel free to provide any additional comments on this question in the text box below:

* 14. During the early stages of treatment, **adult** patients should be monitored for the types of symptoms listed below **once per year**. *Please respond with a tick for each symptom type* (*1 – Strongly disagree; 6 – Strongly agree*). [Slide 25]

	1 – Strongly disagree	2 – Disagree	3 – Somewhat disagree	4 – Somewhat agree	5 – Agree	6 – Strongly agree	Insufficient expertise	Do not wish to answer
Central and peripheral nervous system	○	○	○	○	○	○	○	○
Ocular system	○	○	○	○	○	○	○	○
Cardiovascular system	○	○	○	○	○	○	○	○
Skeletal system	○	○	○	○	○	○	○	○
Pulmonary system	○	○	○	○	○	○	○	○
Enterohepatic system	○	○	○	○	○	○	○	○
Cognitive performance (e.g. learning difficulties)	○	○	○	○	○	○	○	○

Please feel free to provide any additional comments on this question in the text box below:

* 15. Please rank the following examinations and tests in order of their usefulness when monitoring **paediatric** patients receiving CTX treatment (*1=most useful; 5=least useful*). [Slide 26]

	1 – Most useful	2	3	4	5 – Least useful	Insufficient expertise	Do not wish to answer
Cholestanol plasma concentration	○	○	○	○	○	○	○
Brain MRI	○	○	○	○	○	○	○
Neurologic examination (and if necessary neuropsychologic evaluation)	○	○	○	○	○	○	○
Liver function tests	○	○	○	○	○	○	○
Urinary bile alcohol concentration	○	○	○	○	○	○	○

Please feel free to provide any additional comments on this question in the text box below:

* 16. Please rank the following examinations and tests in order of their usefulness when monitoring **adult** patients receiving CTX treatment (*1=most useful; 5=least useful*). [Slide 26]

	1 – Most useful	2	3	4	5 – Least useful	Insufficient expertise	Do not wish to answer
Cholestanol plasma concentration	○	○	○	○	○	○	○
Brain MRI	○	○	○	○	○	○	○
Neurologic examination (and if necessary neuropsychologic evaluation)	○	○	○	○	○	○	○
Liver function tests	○	○	○	○	○	○	○
Urinary bile alcohol concentration	○	○	○	○	○	○	○

Please feel free to provide any additional comments on this question in the text box below:

* 17. **Paediatric** patients should undergo the types of tests listed below**1–2 times per year.** *Please respond with a tick for each test (1 – Strongly disagree; 6 – Strongly agree)*. [Slide 27]

	1 – Strongly disagree	2 – Disagree	3 – Somewhat disagree	4 – Somewhat agree	5 – Agree	6 – Strongly agree	Insufficient expertise	Do not wish to answer
Cholestanol plasma concentration	○	○	○	○	○	○	○	○
Liver function tests	○	○	○	○	○	○	○	○

Please feel free to provide any additional comments on this question in the text box below:

Please specify your level of agreement with the following statements by selecting an option from the dropdown list *(1 – Strongly disagree; 6 – Strongly agree)*.

* 18. **Paediatric** patients should undergo testing for urinary bile alcohol concentrations**once per year.** [Slide 27]

Please feel free to provide any additional comments on this question in the text box below:

* 19. **Paediatric** patients should undergo brain MRI at the time of diagnosis, then **once per year** during follow-up. [Slide 27]

Please feel free to provide any additional comments on this question in the text box below:

* 20. **Paediatric** patients should undergo neurologic (and if necessary neuropsychologic evaluation) testing/examination **twice per year.** [Slide 27]

Please feel free to provide any additional comments on this question in the text box below:

* 21. **Adult** patients should undergo the types of tests/examinations listed below **once per year**. *Please respond with a tick for each type of test/examination* (*1 – Strongly disagree; 6 – Strongly agree*). [Slide 28]

	1 – Strongly disagree	2 – Disagree	3 – Somewhat disagree	4 – Somewhat agree	5 – Agree	6 – Strongly agree	Insufficient expertise	Do not wish to answer
Cholestanol plasma concentration	○	○	○	○	○	○	○	○
Urinary bile alcohol concentration	○	○	○	○	○	○	○	○
Brain MRI	○	○	○	○	○	○	○	○
Neurologic (and if necessary neuropsychologic evaluation)	○	○	○	○	○	○	○	○
Liver function tests	○	○	○	○	○	○	○	○

Please feel free to provide any additional comments on this question in the text box below:

* 22. Disease progression in patients with CTX is better monitored using brain MRI compared with clinical evaluation alone.[5, 6] *Please specify your level of agreement by selecting an option from the dropdown list (1 – Strongly disagree; 6 – Strongly agree).* [Slide 29]

Please feel free to provide any additional comments on this question in the text box below:

23. If you have any additional comments on the questions in this section, please feel free to add them to the following text box:

This Delphi Panel is sponsored by Leadiant Biosciences
Date of preparation: July 2019
GL-NP-1900011

Multidisciplinary care

Please respond with a tick for each type of healthcare professional, for the questions below *(1 – Strongly disagree; 6 – Strongly agree)*.

* 24. The following healthcare professionals are important in the **diagnosis** of **paediatric** patients with CTX. [Slide 31]

	1 – Strongly disagree	2 – Disagree	3 – Somewhat disagree	4 – Somewhat agree	5 – Agree	6 – Strongly agree	Insufficient expertise	Do not wish to answer
Neurologist	○	○	○	○	○	○	○	○
Neuroradiologist	○	○	○	○	○	○	○	○
Paediatrician/Metabolic specialist	○	○	○	○	○	○	○	○
Geneticist	○	○	○	○	○	○	○	○
Ophthalmologist	○	○	○	○	○	○	○	○
Psychiatrist	○	○	○	○	○	○	○	○
Orthopaedic surgeon	○	○	○	○	○	○	○	○
Endocrinologist	○	○	○	○	○	○	○	○
Gastroenterologist	○	○	○	○	○	○	○	○

Please feel free to provide any additional comments on this question in the text box below:

* 25. The following healthcare professionals are important in the **diagnosis** of **adult** patients with CTX. [Slide 32]

	1 – Strongly disagree	2 – Disagree	3 – Somewhat disagree	4 – Somewhat agree	5 – Agree	6 – Strongly agree	Insufficient expertise	Do not wish to answer
Neurologist	○	○	○	○	○	○	○	○
Neuroradiologist	○	○	○	○	○	○	○	○
Metabolic specialist	○	○	○	○	○	○	○	○
Geneticist	○	○	○	○	○	○	○	○
Ophthalmologist	○	○	○	○	○	○	○	○
Psychiatrist	○	○	○	○	○	○	○	○
Orthopaedic surgeon	○	○	○	○	○	○	○	○
Endocrinologist	○	○	○	○	○	○	○	○
Gastroenterologist	○	○	○	○	○	○	○	○
Cardiologist	○	○	○	○	○	○	○	○

Please feel free to provide any additional comments on this question in the text box below:

* 26. The following healthcare professionals should be involved in **prescribing treatment** to **paediatric** patients with CTX. [Slide 33]

	1 – Strongly disagree	2 – Disagree	3 – Somewhat disagree	4 – Somewhat agree	5 – Agree	6 – Strongly agree	Insufficient expertise	Do not wish to answer
Neurologist	○	○	○	○	○	○	○	○
Neuroradiologist	○	○	○	○	○	○	○	○
Paediatrician/Metabolic specialist	○	○	○	○	○	○	○	○
Endocrinologist	○	○	○	○	○	○	○	○
Psychiatrist	○	○	○	○	○	○	○	○
Family doctor	○	○	○	○	○	○	○	○

Please feel free to provide any additional comments on this question in the text box below:

* 27. The following healthcare professionals should be involved in **prescribing treatment** to **adult** patients with CTX. [Slide 34]

	1 – Strongly disagree	2 – Disagree	3 – Somewhat disagree	4 – Somewhat agree	5 – Agree	6 – Strongly agree	Insufficient expertise	Do not wish to answer
Neurologist	○	○	○	○	○	○	○	○
Neuroradiologist	○	○	○	○	○	○	○	○
Metabolic specialist	○	○	○	○	○	○	○	○
Gastroenterologist	○	○	○	○	○	○	○	○
Psychiatrist	○	○	○	○	○	○	○	○
Cardiologist	○	○	○	○	○	○	○	○
Family doctor	○	○	○	○	○	○	○	○
Endocrinologist	○	○	○	○	○	○	○	○
Ophthalmologist	○	○	○	○	○	○	○	○

Please feel free to provide any additional comments on this question in the text box below:

* 28. The following healthcare professionals should be involved in the **follow-up** of **paediatric** patients with CTX. [Slide 35]

	1 – Strongly disagree	2 – Disagree	3 – Somewhat disagree	4 – Somewhat agree	5 – Agree	6 – Strongly agree	Insufficient expertise	Do not wish to answer
Neurologist	○	○	○	○	○	○	○	○
Neuroradiologist	○	○	○	○	○	○	○	○
Paediatrician/Metabolic specialist	○	○	○	○	○	○	○	○
Ophthalmologist	○	○	○	○	○	○	○	○
Family doctor	○	○	○	○	○	○	○	○
Endocrinologist	○	○	○	○	○	○	○	○
Gastroenterologist	○	○	○	○	○	○	○	○
Psychiatrist	○	○	○	○	○	○	○	○

Please feel free to provide any additional comments on this question in the text box below:

* 29. The following healthcare professionals should be involved in the **follow-up** of **adult** patients with CTX. [Slide 36]

	1 – Strongly disagree	2 – Disagree	3 – Somewhat disagree	4 – Somewhat agree	5 – Agree	6 – Strongly agree	Insufficient expertise	Do not wish to answer
Neurologist	○	○	○	○	○	○	○	○
Neuroradiologist	○	○	○	○	○	○	○	○
Ophthalmologist	○	○	○	○	○	○	○	○
Cardiologist	○	○	○	○	○	○	○	○
Gastroenterologist	○	○	○	○	○	○	○	○
Metabolic specialist	○	○	○	○	○	○	○	○
Family doctor	○	○	○	○	○	○	○	○
Endocrinologist	○	○	○	○	○	○	○	○
Psychiatrist	○	○	○	○	○	○	○	○

Please feel free to provide any additional comments on this question in the text box below:

Please respond with a tick for adult and paediatric patients, for the questions below *(1 – Strongly disagree; 6 – Strongly agree)*.

* 30. A **specialist** CTX centre/department should be visited **once per year** by [Slide 37]:

	1 – Strongly disagree	2 – Disagree	3 – Somewhat disagree	4 – Somewhat agree	5 – Agree	6 – Strongly agree	Insufficient expertise	Do not wish to answer
Adult patients with CTX	○	○	○	○	○	○	○	○
Paediatric patients with CTX	○	○	○	○	○	○	○	○

Please feel free to provide any additional comments on this question in the text box below:Other (please specify)

* 31. A **local** CTX centre/department should be visited **twice per year** by [Slide 37]:

	1 – Strongly disagree	2 – Disagree	3 – Somewhat disagree	4 – Somewhat agree	5 – Agree	6 – Strongly agree	Insufficient expertise	Do not wish to answer
Adult patients with CTX	◯	◯	◯	◯	◯	◯	◯	◯
Paediatric patients with CTX	◯	◯	◯	◯	◯	◯	◯	◯

Please feel free to provide any additional comments on this question in the text box below:

32. If you have any additional comments on the questions in this section, please feel free to add them to the following text box:

This Delphi Panel is sponsored by Leadiant Biosciences
Date of preparation: July 2019
GL-NP-1900011

Prognosis

* 33. Please indicate which of the following therapy options improves/stabilises prognosis in the majority of CTX patients.[7-9] *Please respond with a tick for each therapy option (1 – Strongly disagree; 6 – Strongly agree)*. [Slide 39]

	1 – Strongly disagree	2 – Disagree	3 – Somewhat disagree	4 – Somewhat agree	5 – Agree	6 – Strongly agree	Insufficient expertise	Do not wish to answer
CDCA and HMG-CoA reductase inhibitor	○	○	○	○	○	○	○	○
Cholic acid alone	○	○	○	○	○	○	○	○
Cholic acid and HMG-CoA reductase inhibitor	○	○	○	○	○	○	○	○
LDL apheresis	○	○	○	○	○	○	○	○

Please feel free to provide any additional comments on this question in the text box below:

Please specify your level of agreement with the following statements by selecting an option from the dropdown list *(1 – Strongly disagree; 6 – Strongly agree)*.

* 34. There is a positive correlation between the progression of clinical and neuroradiological symptoms in patients with CTX. [Slide 40]

Please feel free to provide any additional comments on this question in the text box below:

* 35. Brain MRI can be used to determine neurological stability in patients with CTX. [Slide 41]

Please feel free to provide any additional comments on this question in the text box below:

* 36. In patients with CTX, the absence of dentate nuclei signal alteration in brain MRI may be an indicator of better prognosis.[10] [Slide 42]

Please feel free to provide any additional comments on this question in the text box below:

* 37. Increased atrophy and/or signal alteration, identified through brain MRI examinations, may be present in patients who have deteriorating neurological symptoms. [Slide 43]

Please feel free to provide any additional comments on this question in the text box below:

38. If you have any additional comments on the questions in this section, please feel free to add them to the following text box:

This Delphi Panel is sponsored by Leadiant Biosciences
Date of preparation: July 2019
GL-NP-1900011

Appendix

This questionnaire uses three different question types to gain consensus; Likert scale, ranking and proportion question types.

Question Type 1: Likert Scale

- You are asked to select your level of agreement with a statement, e.g. “Please specify your level of agreement with the following statements by selecting an option from the dropdown list (1 – Strongly disagree; 6 – Strongly agree)” (Figure 1)
- Consensus definition: ≥70% of participants choose the same option
- If ≥70% of participants respond with ‘1 – Strongly disagree’ or ‘2 – Disagree’, the consensus is disagreement with the specified statement
- If ≥70% of participants respond with ‘5 – Agree’ or ‘6 – Strongly agree’, the consensus is agreement with the specified statement
- If <70% of participant responses select either ‘1 – Strongly disagree/2 – Disagree’ or ‘5 – Agree/6 – Strongly agree’, there is no consensus with the specified statement

Question Type 2: Ranking

- You are asked to rank the options e.g. “Please rank the following factors in order of their impact on treatment outcomes in patients with CTX (1 – Most important; 4 – Least important)”
- Consensus definition: ≥70% of participants chose the same ranking position for individual question options (e.g. ranking position 1)

Question Type 3: Proportion

- You are asked to select the percentage (%) category, that corresponds to the question being asked e.g. “Please indicate the proportion of paediatric patients that present with the following symptoms, prior to a CTX diagnosis (0–24%; 25–49%; 50–74%; 75–100%)”
- Consensus definition: ≥70% of participants chose the same proportion (%) category (e.g. 0–24%)

Please also note the following:

- Any question that a respondent answers with “Insufficient expertise” will not be included in the statistical analysis
- Any question that a respondent answers with “Do not wish to answer” will be included in the statistical analysis:
    - Likert scale: These responses will be considered ‘neutral’ responses (i.e. the respondent neither agrees nor disagrees)
    - Ranking and proportion questions: Respondents will be considered to have not selected the ranking option being analysed

39. Figure 1. Likert Scale

This Delphi Panel is sponsored by Leadiant Biosciences
Date of preparation: July 2019
GL-NP-1900011

References

1. Verrips A, Wevers RA, Van Engelen BG, et al. Effect of simvastatin in addition to chenodeoxycholic acid in patients with cerebrotendinous xanthomatosis. Metabolism 1999;48:233-8.

2. Sekijima Y, Koyama S, Yoshinaga T, et al. Nationwide survey on cerebrotendinous xanthomatosis in Japan. J Hum Genet 2018;63:271-280.

3. Koopman BJ, Wolthers BG, van der Molen JC, et al. Bile acid therapies applied to patients suffering from cerebrotendinous xanthomatosis. Clinica Chimica Acta 1985;152:115-122.

4. Yahalom G, Tsabari R, Molshatzki N, et al. Neurological outcome in cerebrotendinous xanthomatosis treated with chenodeoxycholic acid: early versus late diagnosis. Clinical neuropharmacology 2013;36:78-83.

5. Mascalchi M, Vella A. Neuroimaging Applications in Chronic Ataxias, 2018.

6. Pilo-de-la-Fuente B, Jimenez-Escrig A, Lorenzo J, et al. Cerebrotendinous xanthomatosis in Spain: clinical, prognostic, and genetic survey. European journal of neurology 2011;18:1203-1211.

7. Mignarri A, Rossi S, Ballerini M, et al. Clinical relevance and neurophysiological correlates of spasticity in cerebrotendinous xanthomatosis. Journal of neurology 2011;258:783-790.

8. Mimura Y, Kuriyama M, Tokimura Y, et al. Treatment of cerebrotendinous xanthomatosis with low-density lipoprotein (LDL)-apheresis. J Neurol Sci 1993;114:227-30.

9. Duell PB, Salen G, Eichler FS, et al. Diagnosis, treatment, and clinical outcomes in 43 cases with cerebrotendinous xanthomatosis. Journal of Clinical Lipidology 2018.

10. Mignarri A, Dotti MT, Federico A, et al. The spectrum of magnetic resonance findings in cerebrotendinous xanthomatosis: redefinition and evidence of new markers of disease progression. Journal of neurology 2017;264:862-874.

This Delphi Panel is sponsored by Leadiant Biosciences
Date of preparation: July 2019
GL-NP-1900011

Third Round CTX Delphi Panel Questionnaire

Introduction

Thank you for your participation in Rounds 1 and 2 of the Delphi panel and for taking the time to complete this final Round 3 questionnaire. The aim of the Delphi panel is to achieve consensus on best practices for diagnosing, treating and managing patients with cerebrotendinous xanthomatosis (CTX).

Round 3 Questionnaire Development

Questions that achieved consensus in Round 2 have not been included in Round 3. Questions that did not achieve consensus in Round 2 have been restated again in this Round 3 questionnaire.

In Rounds 1 and 2, some individual questions included multiple question options that you were asked to respond to. For your awareness, any question options that did achieve consensus in Rounds 1 and 2 are presented alongside question options that did not achieve consensus:

- Question options that did achieve consensus in Rounds 1 and 2 are clearly indicated and are not asked again in this questionnaire
- Question options that did not achieve consensus in Rounds 1 and 2 have been restated in Round 3

Based on the analysis of the results from Rounds 1 and 2, one new question has been added to Round 3 (Prognosis; Question 29), and an additional update has been made to the question options in a further question (Diagnosis; Question 4).

Questionnaire Structure and Format

The Round 3 questionnaire includes five sections: Diagnosis, Treatment, Monitoring, Multidisciplinary care and Prognosis. In each section, questions may ask you to respond by ranking options (ranking question type), selecting a percentage category (proportion question type) or selecting your level of agreement with a statement (Likert scale question type). For further information on how the consensus threshold will be applied to the three different question types please see the Appendix.

If you feel that you do not have sufficient expertise to answer an individual question, please select ‘Insufficient expertise’. Alternatively, if you do not wish to answer an individual question for any other reason, please select ‘Do not wish to answer’. If you would like to provide justification for your answers, or have any additional comments, please feel free to complete the available text boxes at the end of each question or section.

The responses and comments you provide throughout this questionnaire will be considered when writing up the final results of the Delphi panel.

Completing the Questionnaire

Please provide responses to all questions based on what you **believe to be best practice**.

In line with Delphi methodology, all participants should complete this Round 3 questionnaire taking into account the group results from the previous rounds, to provide you with an opportunity to reassess your initial judgements. In this Round 3 questionnaire we refer to slides included in the Round 2 Results Summary slideset, which is attached to your Round 3 invitation email. Therefore, **we recommend that you review this slideset while completing the Round 3 questionnaire**, relevant slide numbers have been added at the end of each question/statement.

Please note this Round 3 questionnaire should take up to 15–20 minutes to complete, and your responses will remain anonymous to the other Delphi panel participants. The questionnaire must be completed in a single sitting and responses will not be saved until you have finished the entire survey.

This Delphi Panel is sponsored by Leadiant Biosciences
Date of preparation: December 2019
GL-NP-1900016

Third Round CTX Delphi Panel Questionnaire

Adverse Event Reporting

Should you raise an adverse event/product complaint associated with the use of a Leadiant Biosciences medicinal product in a specific patient or group of patients, we will need to report this, even if it has already been reported by you directly to the company or the regulatory authorities using the MHRA's 'Yellow Card' system. If you decide to disclose your personal details in association with any adverse event/product complaint report, this information will be disclosed to the commissioning company. In such a situation you may be contacted specifically in relation to that adverse event. Everything else you contribute to the questionnaire will continue to remain confidential.

* 1. **Please tick the box to confirm that you agree for your details to be passed on to Leadiant Biosciences should an adverse event/product complaint associated with the use of a Leadiant Biosciences medicinal product be recorded.**

☐ Yes

This Delphi Panel is sponsored by Leadiant Biosciences
Date of preparation: December 2019
GL-NP-1900016

Third Round CTX Delphi Panel Questionnaire

Questionnaire

Please note the following definitions that apply throughout the questionnaire:

- ***Adult patients with CTX: aged ≥18 years***
- ***Paediatric patients with CTX: aged <18 years***

This Delphi Panel is sponsored by Leadiant Biosciences
Date of preparation: December 2019
GL-NP-1900016

Diagnosis

* 2. *When answering this question, consider a scenario where a patient has been referred to you following the onset of symptoms.*

You were previously asked to rank five indicators in order of which has the greatest diagnostic value, when considering a CTX diagnosis *(1=greatest diagnostic value; 5=least diagnostic value)*. [Slide 12]. The indicator displayed below reached consensus with respect to the following ranking position:

- *CYP27A1* genetic mutation: Ranking position 1

Please rank the remaining indicators in order of which has the greatest diagnostic value, when considering a CTX diagnosis (ranking positions 2–5)

	2	3	4	5	Insufficient expertise	Do not wish to answer
An affected sibling	○	○	○	○	○	○
Clinical signs and symptoms	○	○	○	○	○	○
Biochemical pathogenesis (assessed through biochemical testing)	○	○	○	○	○	○
Brain MRI findings	○	○	○	○	○	○

Please feel free to provide any additional comments on this question in the text box below:

* 3. You were previously asked to rank five tests/examinations in order of importance when confirming a CTX diagnosis *(1=most important; 5=least important)*. [Slide 14]. The tests/examinations displayed below reached consensus with respect to the following ranking positions:

- Genetic testing alone: Ranking position 1
- Determination of serum cholestanol levels: Ranking position 2

Please rank the remaining tests/examinations in order of importance when confirming a CTX diagnosis (ranking positions 3–5)

	3	4	5	Insufficient expertise	Do not wish to answer
Detection of urinary bile alcohols	○	○	○	○	○
Determination of plasma bile acids (mainly cholic acid and chenodeoxycholic acid)	○	○	○	○	○
Conventional brain MRI	○	○	○	○	○

Please feel free to provide any additional comments on this question in the text box below:

* 4. **Please indicate the proportion of <u>paediatric</u> patients that present with the following symptoms, prior to a CTX diagnosis.** [Slide 16] ***(Please respond with a tick for each symptom type)***

	0–24%	25–49%	50–74%	75–100%	Insufficient expertise	Do not wish to answer
Early psychiatric symptoms (e.g. autism)	◯	◯	◯	◯	◯	◯
Tendon xanthomas	◯	◯	◯	◯	◯	◯
Neonatal cholestatic jaundice	◯	◯	◯	◯	◯	◯
Cerebellar system findings (e.g. ataxia symptoms and tremor)	◯	◯	◯	◯	◯	◯
Epilepsy	◯	◯	◯	◯	◯	◯
Peripheral neuropathy	◯	◯	◯	◯	◯	◯
Z-scores below the expected range for age in bone mineral density (BMD)	◯	◯	◯	◯	◯	◯

Please note that in Round 1, participants were in consensus that paediatric patients presented with the following symptoms, prior to a CTX diagnosis (therefore these options have not been included in the table above)

- Chronic diarrhoea: Consensus agreement
- Bilateral juvenile cataracts: Consensus agreement
- Mental retardation (e.g. learning difficulties): Consensus agreement

Please feel free to provide any additional comments on this question in the text box below:

* 5. **Please indicate the proportion of <u>adult</u> patients that present with the following symptoms, prior to a CTX diagnosis.** [Slide 18] ***(Please respond with a tick for each symptom type)***

The symptom displayed below reached consensus in the previous round with the following proportion:

- Early-onset dementia: 25–49%

	0–24%	25–49%	50–74%	75–100%	Insufficient expertise	Do not wish to answer
Early-onset movement disorder (e.g. atypical parkinsonism)	◯	◯	◯	◯	◯	◯
Epilepsy	◯	◯	◯	◯	◯	◯

Please note that in Round 1, participants were in consensus that adult patients presented with the following symptoms, prior to a CTX diagnosis (therefore these options have not been included in the table above)

- Infantile-onset diarrhoea: Consensus agreement
- Childhood-onset cataracts: Consensus agreement
- Tendon xanthomas: Consensus agreement
- Psychiatric symptoms: Consensus agreement
- Peripheral neuropathy: Consensus agreement
- Cerebellar signs: Consensus agreement
- Pyramidal signs: Consensus agreement

Please feel free to provide any additional comments on this question in the text box below:

6. If you have any additional comments on the questions in this section, please feel free to add them to the following text box:

This Delphi Panel is sponsored by Leadiant Biosciences
Date of preparation: December 2019
GL-NP-1900016

Treatment

* 7. You were previously asked to rank five factors in order of their impact on treatment outcomes in patients with CTX *(1=greatest impact; 5=least impact)*. [Slide 23]. The factors displayed below reached consensus with respect to the following ranking positions:

- Age at diagnosis and treatment initiation: Ranking position 1
- Extent of neurological deterioration: Ranking position 2

Please rank the remaining factors in order of their impact on treatment outcomes in patients with CTX (ranking positions 3–5)

	3	4	5	Insufficient expertise	Do not wish to answer
Cholestanol level at diagnosis	◯	◯	◯	◯	◯
Treatment compliance	◯	◯	◯	◯	◯
Characteristics of cerebellar signal abnormalities	◯	◯	◯	◯	◯

Please feel free to provide any additional comments on this question in the text box below:

* 8. **Please rank the following parameters in order of their usefulness for measuring treatment efficacy in patients with CTX *(1=most useful; 5=least useful).*** [Slide 25]

	1	2	3	4	5	Insufficient expertise	Do not wish to answer
Levels of serum cholestanol alone	◯	◯	◯	◯	◯	◯	◯
Clinical presentation/neurological examination	◯	◯	◯	◯	◯	◯	◯
Brain MRI	◯	◯	◯	◯	◯	◯	◯
Levels of urinary bile alcohols	◯	◯	◯	◯	◯	◯	◯
Electrophysiological examinations (e.g. electromyography, nerve conduction velocity, electroencephalography)	◯	◯	◯	◯	◯	◯	◯

Please feel free to provide any additional comments on this question in the text box below:

* 9. You were previously asked to rank five therapy options in order of their effectiveness for treating the underlying biochemical abnormalities in CTX *(1=most effective; 5=least effective)*.[1-3] [Slide 27]. The therapy options displayed below reached consensus with respect to the following ranking positions:

- CDCA alone: Ranking position 1
- LDL apheresis: Ranking position 5

Please rank the remaining therapy options in order of their effectiveness for treating the underlying biochemical abnormalities in CTX (ranking positions 2–4)

	2	3	4	Insufficient expertise	Do not wish to answer
CDCA and HMG-CoA reductase inhibitor	◯	◯	◯	◯	◯
Cholic acid alone	◯	◯	◯	◯	◯
Cholic acid and HMG-CoA reductase inhibitor	◯	◯	◯	◯	◯

Please feel free to provide any additional comments on this question in the text box below:

* 10. **Research indicates that treating CTX mothers with CDCA during pregnancy acts as an important means of protection against damage to the fetus and miscarriage. Please specify your level of agreement by selecting an option from the dropdown list *(1 – Strongly disagree; 6 – Strongly agree).***[4] [Slide 31]

Please feel free to provide any additional comments on this question in the text box below:

11. If you have any additional comments on the questions in this section, please feel free to add them to the following text box:

This Delphi Panel is sponsored by Leadiant Biosciences
Date of preparation: December 2019
GL-NP-1900016

Monitoring

In this section, please consider adult and paediatric patients diagnosed with CTX who are currently receiving CDCA, unless otherwise specified.

* 12. **During the early stages of treatment, paediatric patients should be monitored for the types of symptoms listed below 1–2 times per year.** ***Please respond with a tick for each symptom type*** **(*1 – Strongly disagree; 6 – Strongly agree*).** [Slide 37]

Please consider the following results which reached consensus in the previous round when answering this question:

- Central and peripheral nervous system: Consensus agreement
- Ocular system: Consensus agreement
- Enterohepatic system: Consensus agreement
- Cognitive performance (e.g. learning difficulties): Consensus agreement

	1 – Strongly disagree	2 – Disagree	3 – Somewhat disagree	4 – Somewhat agree	5 – Agree	6 – Strongly agree	Insufficient expertise	Do not wish to answer
Cardiovascular system	○	○	○	○	○	○	○	○
Skeletal system	○	○	○	○	○	○	○	○
Pulmonary system	○	○	○	○	○	○	○	○

Please feel free to provide any additional comments on this question in the text box below:

* 13. **During the early stages of treatment, <u>adult</u> patients should be monitored for the types of symptoms listed below <u>once per year</u>. *Please respond with a tick for the symptom type* (*1 – Strongly disagree; 6 – Strongly agree*).** [Slide 39]

Please consider the following results which reached consensus in the previous round when answering this question:

- Central and peripheral nervous system: Consensus agreement
- Ocular system: Consensus agreement
- Cardiovascular system: Consensus agreement
- Skeletal system: Consensus agreement
- Enterohepatic system: Consensus agreement
- Cognitive performance (e.g. learning difficulties): Consensus agreement

	1 – Strongly disagree	2 – Disagree	3 – Somewhat disagree	4 – Somewhat agree	5 – Agree	6 – Strongly agree	Insufficient expertise	Do not wish to answer
Pulmonary system	○	○	○	○	○	○	○	○

Please feel free to provide any additional comments on this question in the text box below:

* 14. You were previously asked to rank five examinations/tests in order of their usefulness when monitoring <u>paediatric</u> patients receiving CTX treatment *(1=most useful; 5=least useful)*. [Slide 41]. The test displayed below reached consensus with respect to the following ranking position:

- Cholestanol plasma concentration: Ranking position 1

Please rank the remaining examinations/tests in order of their usefulness when monitoring <u>paediatric</u> patients receiving CTX treatment (ranking positions 2–5)

	2	3	4	5	Insufficient expertise	Do not wish to answer
Brain MRI	○	○	○	○	○	○
Neurologic examination (and if necessary neuropsychologic evaluation)	○	○	○	○	○	○
Liver function tests	○	○	○	○	○	○
Urinary bile alcohol concentration	○	○	○	○	○	○

Please feel free to provide any additional comments on this question in the text box below:

* 15. You were previously asked to rank five examinations/tests in order of their usefulness when monitoring <u>adult</u> patients receiving CTX treatment *(1=most useful; 5=least useful)*. [Slide 43]. The test displayed below reached consensus with respect to the following ranking position:

- Cholestanol plasma concentration: Ranking position 1

Please rank the remaining examinations/tests in order of their usefulness when monitoring <u>adult</u> patients receiving CTX treatment (ranking positions 2–5)

	2	3	4	5	Insufficient expertise	Do not wish to answer
Brain MRI	○	○	○	○	○	○
Neurologic examination (and if necessary neuropsychologic evaluation)	○	○	○	○	○	○
Liver function tests	○	○	○	○	○	○
Urinary bile alcohol concentration	○	○	○	○	○	○

Please feel free to provide any additional comments on this question in the text box below:

Please specify your level of agreement with the following statements by selecting an option from the dropdown list *(1 – Strongly disagree; 6 – Strongly agree)*.

* 16. **<u>Paediatric</u> patients should undergo testing for urinary bile alcohol concentrations <u>once per year</u>.** [Slide 47]

Please feel free to provide any additional comments on this question in the text box below:

* 17. **<u>Paediatric</u> patients should undergo brain MRI at the time of diagnosis, then <u>once per year</u> during follow-up.** [Slide 49]

Please feel free to provide any additional comments on this question in the text box below:

* 18. **<u>Adult</u> patients should undergo the types of tests/examinations listed below <u>once per year</u>. *Please respond with a tick for each type of test/examination* (*1 – Strongly disagree; 6 – Strongly agree*).** [Slide 53]

Please consider the following results which reached consensus in the previous round when answering this question:

- Cholestanol plasma concentration: Consensus agreement
- Neurologic (and if necessary neuropsychologic evaluation): Consensus agreement
- Liver function tests: Consensus agreement

	1 – Strongly disagree	2 – Disagree	3 – Somewhat disagree	4 – Somewhat agree	5 – Agree	6 – Strongly agree	Insufficient expertise	Do not wish to answer
Urinary bile alcohol concentration	◯	◯	◯	◯	◯	◯	◯	◯
Brain MRI	◯	◯	◯	◯	◯	◯	◯	◯

Please feel free to provide any additional comments on this question in the text box below:

* 19. **Disease progression in patients with CTX is better monitored using brain MRI compared with clinical evaluation alone. *Please specify your level of agreement by selecting an option from the dropdown list (1 – Strongly disagree; 6 – Strongly agree).*** [Slide 55][5,6]

Please feel free to provide any additional comments on this question in the text box below:

20. If you have any additional comments on the questions in this section, please feel free to add them to the following text box:

This Delphi Panel is sponsored by Leadiant Biosciences
Date of preparation: December 2019
GL-NP-1900016

Multidisciplinary care

Please specify your level of agreement with the following statements by responding with a tick for each type of healthcare professional (1 – Strongly disagree; 6 – Strongly agree).

* 21. **The following healthcare professionals are important in the diagnosis of paediatric patients with CTX.** [Slides 62–63]

Please consider the following results which reached consensus in the previous round when answering this question:

- Neurologist: Consensus agreement
- Paediatrician/Metabolic specialist: Consensus agreement
- Geneticist: Consensus agreement

	1 – Strongly disagree	2 – Disagree	3 – Somewhat disagree	4 – Somewhat agree	5 – Agree	6 – Strongly agree	Insufficient expertise	Do not wish to answer
Neuroradiologist	◯	◯	◯	◯	◯	◯	◯	◯
Ophthalmologist	◯	◯	◯	◯	◯	◯	◯	◯
Psychiatrist	◯	◯	◯	◯	◯	◯	◯	◯
Orthopaedic surgeon	◯	◯	◯	◯	◯	◯	◯	◯
Endocrinologist	◯	◯	◯	◯	◯	◯	◯	◯
Gastroenterologist	◯	◯	◯	◯	◯	◯	◯	◯

Please feel free to provide any additional comments on this question in the text box below:

* 22. **The following healthcare professionals are important in the <u>diagnosis</u> of <u>adult</u> patients with CTX.** [Slides 65–66]

Please consider the following results which reached consensus in the previous round when answering this question:

- Neurologist: Consensus agreement
- Metabolic specialist: Consensus agreement
- Geneticist: Consensus agreement

	1 – Strongly disagree	2 – Disagree	3 – Somewhat disagree	4 – Somewhat agree	5 – Agree	6 – Strongly agree	Insufficient expertise	Do not wish to answer
Neuroradiologist	○	○	○	○	○	○	○	○
Ophthalmologist	○	○	○	○	○	○	○	○
Psychiatrist	○	○	○	○	○	○	○	○
Orthopaedic surgeon	○	○	○	○	○	○	○	○
Endocrinologist	○	○	○	○	○	○	○	○
Gastroenterologist	○	○	○	○	○	○	○	○
Cardiologist	○	○	○	○	○	○	○	○

Please feel free to provide any additional comments on this question in the text box below:

* 23. **The following healthcare professionals should be involved in <u>prescribing treatment</u> to <u>paediatric</u> patients with CTX.** [Slide 68]

Please consider the following results which reached consensus in the previous round when answering this question:

- Neurologist: Consensus agreement
- Neuroradiologist: Consensus disagreement
- Paediatrician/Metabolic specialist: Consensus agreement
- Family doctor: Consensus disagreement

	1 – Strongly disagree	2 – Disagree	3 – Somewhat disagree	4 – Somewhat agree	5 – Agree	6 – Strongly agree	Insufficient expertise	Do not wish to answer
Endocrinologist	○	○	○	○	○	○	○	○
Psychiatrist	○	○	○	○	○	○	○	○

Please feel free to provide any additional comments on this question in the text box below:

* 24. **The following healthcare professionals should be involved in <u>prescribing treatment</u> to <u>adult</u> patients with CTX.** [Slides 70–71]

Please consider the following results which reached consensus in the previous round when answering this question:

- Neurologist: Consensus agreement
- Neuroradiologist: Consensus disagreement
- Metabolic specialist: Consensus agreement
- Cardiologist: Consensus disagreement
- Family doctor: Consensus disagreement
- Ophthalmologist: Consensus disagreement

	1 – Strongly disagree	2 – Disagree	3 – Somewhat disagree	4 – Somewhat agree	5 – Agree	6 – Strongly agree	Insufficient expertise	Do not wish to answer
Gastroenterologist	◯	◯	◯	◯	◯	◯	◯	◯
Psychiatrist	◯	◯	◯	◯	◯	◯	◯	◯
Endocrinologist	◯	◯	◯	◯	◯	◯	◯	◯

Please feel free to provide any additional comments on this question in the text box below:

* 25. **The following healthcare professionals should be involved in the <u>follow-up</u> of <u>paediatric</u> patients with CTX.** [Slides 73–74]

Please consider the following results which reached consensus in the previous round when answering this question:

- Neurologist: Consensus agreement
- Paediatrician/Metabolic specialist: Consensus agreement

	1 – Strongly disagree	2 – Disagree	3 – Somewhat disagree	4 – Somewhat agree	5 – Agree	6 – Strongly agree	Insufficient expertise	Do not wish to answer
Neuroradiologist	○	○	○	○	○	○	○	○
Ophthalmologist	○	○	○	○	○	○	○	○
Family doctor	○	○	○	○	○	○	○	○
Endocrinologist	○	○	○	○	○	○	○	○
Gastroenterologist	○	○	○	○	○	○	○	○
Psychiatrist	○	○	○	○	○	○	○	○

Please feel free to provide any additional comments on this question in the text box below:

* 26. **The following healthcare professionals should be involved in the <u>follow-up</u> of <u>adult</u> patients with CTX.** [Slides 76–77]

Please consider the following results which reached consensus in the previous round when answering this question:

- Neurologist: Consensus agreement
- Ophthalmologist: Consensus agreement
- Metabolic specialist: Consensus agreement

	1 – Strongly disagree	2 – Disagree	3 – Somewhat disagree	4 – Somewhat agree	5 – Agree	6 – Strongly agree	Insufficient expertise	Do not wish to answer
Neuroradiologist	◯	◯	◯	◯	◯	◯	◯	◯
Cardiologist	◯	◯	◯	◯	◯	◯	◯	◯
Gastroenterologist	◯	◯	◯	◯	◯	◯	◯	◯
Family doctor	◯	◯	◯	◯	◯	◯	◯	◯
Endocrinologist	◯	◯	◯	◯	◯	◯	◯	◯
Psychiatrist	◯	◯	◯	◯	◯	◯	◯	◯

Please feel free to provide any additional comments on this question in the text box below:

27. If you have any additional comments on the questions in this section, please feel free to add them to the following text box:

This Delphi Panel is sponsored by Leadiant Biosciences
Date of preparation: December 2019
GL-NP-1900016

Prognosis

* 28. **Please indicate which of the following therapy options improves/stabilises prognosis in the majority of CTX patients. *Please respond with a tick for each therapy option (1 – Strongly disagree; 6 – Strongly agree).*** [Slide 86][7-9]

Please consider the following results which reached consensus in the previous round when answering this question:

- CDCA alone: Consensus agreement
- CDCA and HMG-CoA reductase inhibitor: Consensus agreement
- LDL apheresis: Consensus disagreement

	1 – Strongly disagree	2 – Disagree	3 – Somewhat disagree	4 – Somewhat agree	5 – Agree	6 – Strongly agree	Insufficient expertise	Do not wish to answer
Cholic acid alone	◯	◯	◯	◯	◯	◯	◯	◯
Cholic acid and HMG-CoA reductase inhibitor	◯	◯	◯	◯	◯	◯	◯	◯

Please feel free to provide any additional comments on this question in the text box below:

Please specify your level of agreement with the following statements by selecting an option from the dropdown list *(1 – Strongly disagree; 6 – Strongly agree)*.

* 29. **CDCA alone is a preferred first line treatment compared to CDCA and HMG-CoA reductase inhibitor for treating the underlying biochemical abnormalities in CTX.** [New question]

Please feel free to provide any additional comments on this question in the text box below:

* 30. **There is a positive correlation between the progression of clinical and neuroradiological symptoms in patients with CTX.** [Slide 88]

Please feel free to provide any additional comments on this question in the text box below:

* 31. **Brain MRI can be used to determine neurological stability in patients with CTX.** [Slide 90]

Please feel free to provide any additional comments on this question in the text box below:

32. If you have any additional comments on the questions in this section, please feel free to add them to the following text box:

This Delphi Panel is sponsored by Leadiant Biosciences
Date of preparation: December 2019
GL-NP-1900016

Third Round CTX Delphi Panel Questionnaire

Appendix

This questionnaire uses three different question types to gain consensus; Likert scale, ranking and proportion question types.

Question Type 1: Likert Scale

- You are asked to select your level of agreement with a statement, e.g. “Please specify your level of agreement with the following statements by selecting an option from the dropdown list (1 – Strongly disagree; 6 – Strongly agree)” (Figure 1)
- Consensus definition: ≥70% of participants choose the same option
- If ≥70% of participants respond with ‘1 – Strongly disagree’ or ‘2 – Disagree’, the consensus is disagreement with the specified statement
- If ≥70% of participants respond with ‘5 – Agree’ or ‘6 – Strongly agree’, the consensus is agreement with the specified statement
- If <70% of participant responses select either ‘1 – Strongly disagree/2 – Disagree’ or ‘5 – Agree/6 – Strongly agree’, there is no consensus with the specified statement

Question Type 2: Ranking

- You are asked to rank the options e.g. “Please rank the following factors in order of their impact on treatment outcomes in patients with CTX (1 – Most important; 4 – Least important)”
- Consensus definition: ≥70% of participants chose the same ranking position for individual question options (e.g. ranking position 1)

Question Type 3: Proportion

- You are asked to select the percentage (%) category, that corresponds to the question being asked e.g. “Please indicate the proportion of paediatric patients that present with the following symptoms, prior to a CTX diagnosis (0–24%; 25–49%; 50–74%; 75–100%)”
- Consensus definition: ≥70% of participants chose the same proportion (%) category (e.g. 0–24%)

Please also note the following:

- Any question that a respondent answers with “Insufficient expertise” will not be included in the statistical analysis
- Any question that a respondent answers with “Do not wish to answer” will be included in the statistical analysis:
  - Likert scale: These responses will be considered ‘neutral’ responses (i.e. the respondent neither agrees nor disagrees)
  - Ranking and proportion questions: Respondents will be considered to have not selected the ranking option being analysed

33. Figure 1. Likert Scale

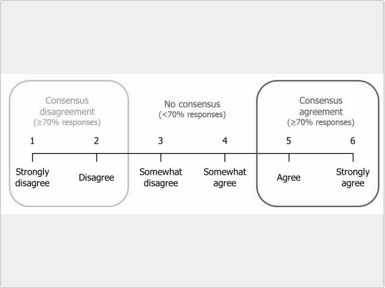

This Delphi Panel is sponsored by Leadiant Biosciences
Date of preparation: December 2019
GL-NP-1900016

References

1. Verrips A, Wevers RA, Van Engelen BG, et al. Effect of simvastatin in addition to chenodeoxycholic acid in patients with cerebrotendinous xanthomatosis. Metabolism 1999;48:233-8.

2. Sekijima Y, Koyama S, Yoshinaga T, et al. Nationwide survey on cerebrotendinous xanthomatosis in Japan. J Hum Genet 2018;63:271-280.

3. Koopman BJ, Wolthers BG, van der Molen JC, et al. Bile acid therapies applied to patients suffering from cerebrotendinous xanthomatosis. Clinica Chimica Acta 1985;152:115-122.

4. Yahalom G, Tsabari R, Molshatzki N, et al. Neurological outcome in cerebrotendinous xanthomatosis treated with chenodeoxycholic acid: early versus late diagnosis. Clinical neuropharmacology 2013;36:78-83.

5. Mascalchi M, Vella A. Neuroimaging Applications in Chronic Ataxias, 2018.

6. Pilo-de-la-Fuente B, Jimenez-Escrig A, Lorenzo J, et al. Cerebrotendinous xanthomatosis in Spain: clinical, prognostic, and genetic survey. European journal of neurology 2011;18:1203-1211.

7. Mignarri A, Rossi S, Ballerini M, et al. Clinical relevance and neurophysiological correlates of spasticity in cerebrotendinous xanthomatosis. Journal of neurology 2011;258:783-790.

8. Mimura Y, Kuriyama M, Tokimura Y, et al. Treatment of cerebrotendinous xanthomatosis with low-density lipoprotein (LDL)-apheresis. J Neurol Sci 1993;114:227-30.

9. Duell PB, Salen G, Eichler FS, et al. Diagnosis, treatment, and clinical outcomes in 43 cases with cerebrotendinous xanthomatosis. Journal of Clinical Lipidology 2018.

This Delphi Panel is sponsored by Leadiant Biosciences
Date of preparation: December 2019
GL-NP-1900016
