# Supplementary material for: Expert opinion on diagnosing, treating and managing patients with cerebrotendinous xanthomatosis (CTX): a modified Delphi study
Source: Orphanet J Rare Dis. 2021 Aug 6;16:353. doi: 10.1186/s13023-021-01980-5 (PMC8349076; doi:10.1186/s13023-021-01980-5)
Supplement: Supplementary file 3 — Additional file 3. Table S1 - TLR search terms. [file 13023_2021_1980_MOESM3_ESM.pdf]

Supplementary Table 1. TLR search terms

Search Term Group	Number	Search Terms	TLR Results	TLR Update Results (Embase/MEDLINE)
**Cerebrotendinous xanthomatosis**	1	"cerebrotendin?ous xanthomatosis".tw.	1368	765/630
	2	(cerebr* adj (cholesterosis or cholesterinosis)).tw.	3	1/1
	3	"van Bogaert-Scherer-Epstein".tw.	11	8/5
	4	"Sterol 27-hydroxylase deficien$".tw.	32	17/15
	5	(Cholestanol$ adj (lipidosis or storage)).tw.	6	2/3
	6	(Thi?baut$ syndrome).tw.	3	1/1
	7	Or/1-6	1383	772/638
**Exclusion terms**	8	exp animals/ not exp humans/	9168178	4332509/4514185
	9	("conference abstract" or "conference review" or comment or letter or editorial).pt.	6236002	4848266/1671196
	10	8 or 9	15034246	8843639/6123276
**Combined**	11	7 not 10	1178	610/577
**TLR limits**	12	Remove duplicates from 11	**664**	-
**TLR update limits**	13	Limit 11 to dd=20180510-20181113 (Embase; 10th May 2018–13th November 2018)	-	**8** (Embase)
		Limit 11 to yr=2018-current (MEDLINE; 1st January 2018– 13th November 2018)	-	**25** (MEDLINE)

TLR searches (rows 1–12) run on 18th May 2018. Embase 1974 to 2018 May 17, Ovid MEDLINE(R) Epub Ahead of Print, In-Process & Other Non-Indexed Citations, Ovid MEDLINE(R) Daily and Ovid MEDLINE(R) 1946 to Present. TLR update searches (rows 1–11 and row 13) run on 13th November 2018. Embase 1974 to 2018 November 12, Ovid MEDLINE(R) Epub Ahead of Print, In-Process & Other Non-Indexed Citations, Ovid MEDLINE(R) Daily and Ovid MEDLINE(R) 1946 to 2018 November 12. TLR: targeted literature review.
