# Supplementary material for: Expert opinion on diagnosing, treating and managing patients with cerebrotendinous xanthomatosis (CTX): a modified Delphi study
Source: Orphanet J Rare Dis. 2021 Aug 6;16:353. doi: 10.1186/s13023-021-01980-5 (PMC8349076; doi:10.1186/s13023-021-01980-5)
Supplement: Supplementary file 4 — Additional file 4. Table S2 - TLR eligibility criteria. [file 13023_2021_1980_MOESM4_ESM.pdf]

Supplementary Table 2. TLR eligibility criteria

Search Term Group	Inclusion Criteria	Exclusion Criteria
**Population**	• Patients with CTX<br>• Any individual undergoing testing for CTX	• Patients without CTX<br>• Individuals not undergoing testing for CTX
**Interventions**	• Any or none	• N/A
**Comparators**	• Any or none	• N/A
**Outcomes**	*Outcomes: Screening, diagnosis and confirmation of CTX*	
	Articles reporting outcomes related to CTX testing (in a diagnostic or confirmatory setting) including but not limited to:<br>• Biochemical testing<br>• Molecular genetic testing for *CYP27A1* pathogenic variants<br>• Pharmacokinetic (PK)/pharmacodynamic (PD) methods and other laboratory findings<br>• Brain MRI or computerized tomography (CT) showing atrophy or lesions<br>• Magnetic resonance (MR) spectroscopy showing increased brain lactate concentration<br>• Other symptom-based diagnoses, including:<br>• Infantile-onset diarrhoea<br>• Childhood-onset cataract<br>• Young adult-onset tendon xanthomas<br>• Adult-onset progression neurologic dysfunction, such as dementia or seizures	• Articles not reporting any eligible outcomes related to the screening, diagnosis or confirmation of CTX

Search Term Group	Inclusion Criteria	Exclusion Criteria
	Outcomes: Treatment	
	Articles reporting at least one of the following outcomes when assessing an established or potential treatment for CTX:<br>• Prophylactic CTX treatment, early CTX treatment or treatment to manage CTX<br>• Clinical outcomes (efficacy, safety and tolerability)<br>• Quality of life outcomes (utility values and quality of life)	Articles not reporting any eligible outcomes relating to the treatment of CTX
	Outcomes: Monitoring	
	Articles reporting on outcomes related to monitoring of patients with CTX, including:<br>• Neurologic and neuropsychological evaluation<br>• Brain MRI<br>• Echocardiogram<br>• Total body density<br>• Genetic counselling<br>• Follow-up appointments	• Articles not reporting any eligible outcomes related to monitoring of patients with CTX
	Outcomes: Multidisciplinary care	
	Articles reporting on multiple care settings or multidisciplinary teams (MDTs) caring for CTX patients, including:<br>• Healthcare professionals in MDTs<br>• Care received by MDTs<br>• CTX departments or clinics	• Articles not reporting any eligible outcomes related to multidisciplinary care for patients with CTX

Search Term Group	Inclusion Criteria	Exclusion Criteria
	Outcomes: Prognosis	
	Articles reporting on outcomes related to prognosis of patients with CTX, including:<br>• Worsening/stability of symptoms<br>• Patients who have not responded to treatment	• Articles not reporting any eligible outcomes related to prognosis of patients with CTX
**Study design**	• Randomised controlled trials<br>• Non-randomised interventional studies<br>• Observational studies (including registries and surveys)<br>• Case reports or series (included separately at abstract stage but not taken forward to full-text stage)<br>• Cross-sectional studies<br>• Narrative reviews	• Congress abstracts or congress reviews<br>• Commentaries or letters<br>• Editorials<br>• Economic evaluations
**Date**	• Database inception to present	• N/A
**Other considerations**	• Only abstracts or full-texts in the English language will be included<br>• Studies conducted in humans	• Abstracts or full-texts not in the English language<br>• *In vitro* or animal studies

Each title and abstract were reviewed against the inclusion/exclusion criteria by one reviewer, with a second independent reviewer validating 10% of full-text articles deemed suitable for inclusion. CT: computed tomography; CTX: cerebrotendinous xanthomatosis; MDT: multidisciplinary team; MR: magnetic resonance; MRI: magnetic resonance imaging; N/A: not applicable; PK/PD: pharmacokinetics/pharmacodynamics.
