# Supplementary material for: Expert opinion on diagnosing, treating and managing patients with cerebrotendinous xanthomatosis (CTX): a modified Delphi study
Source: Orphanet J Rare Dis. 2021 Aug 6;16:353. doi: 10.1186/s13023-021-01980-5 (PMC8349076; doi:10.1186/s13023-021-01980-5)
Supplement: Supplementary file 5 — Additional file 5. Table S3 - Full-texts prioritised from the TLR to inform Delphi panel questionnaires. [file 13023_2021_1980_MOESM5_ESM.pdf]

Supplementary Table 3. Full-texts prioritised from the TLR to inform Delphi panel questionnaires

Author (Year)	Title
Amador *et al.* (2018)	Treatment with chenodeoxycholic acid in cerebrotendinous xanthomatosis: clinical, neurophysiological, and quantitative brain structural outcomes
Batta *et al.* (2004)	Hydrophilic 7β-hydroxy bile acids, lovastatin, and cholestyramine are ineffective in the treatment of cerebrotendinous xanthomatosis
Beppu *et al.* (1982)	Serum bile acid profiles in cerebrotendinous xanthomatosis
Berginer *et al.* (1982)	EEG in cerebrotendinous xanthomatosis (CTX)
Berginer *et al.* (1984)	Long-term treatment of cerebrotendinous xanthomatosis with chenodeoxycholic acid
Bleyle *et al.* (2016)	Update on newborn dried bloodspot testing for cerebrotendinous xanthomatosis: an available high-throughput liquid-chromatography tandem mass spectrometry method
DeBarber *et al.* (2018)	Newborn screening for cerebrotendinous xanthomatosis is the solution for early identification and treatment
DeBarber *et al.* (2014)	A blood test for cerebrotendinous xanthomatosis with potential for disease detection in newborns
Duell *et al.* (2018)	Diagnosis, treatment, and clinical outcomes in 43 cases with cerebrotendinous xanthomatosis
Ginanneschi *et al.* (2013)	Polyneuropathy in cerebrotendinous xanthomatosis and response to treatment with chenodeoxycholic acid
Inglese *et al.* (2003)	Quantification of brain damage in cerebrotendinous xanthomatosis with magnetization transfer MR imaging
Köhler *et al.* (2018)	Adulthood leukodystrophies
Mascalchi and Vella. (2018)	Neuroimaging Applications in Chronic Ataxias
Mignarri *et al.* (2017)	The spectrum of magnetic resonance findings in cerebrotendinous xanthomatosis: redefinition and evidence of new markers of disease progression
Mignarri *et al.* (2014)	A suspicion index for early diagnosis and treatment of cerebrotendinous xanthomatosis

Author (Year)	Title
Mignarri *et al.* (2016)	Evaluation of cholesterol metabolism in cerebrotendinous xanthomatosis
Mignarri *et al.* (2011)	Clinical relevance and neurophysiological correlates of spasticity in cerebrotendinous xanthomatosis
Nie *et al.* (2014)	Cerebrotendinous xanthomatosis: a comprehensive review of pathogenesis, clinical manifestations, diagnosis, and management
Pilo-de-la-Fuente *et al.* (2011)	Cerebrotendinous xanthomatosis in Spain: clinical, prognostic, and genetic survey
Pitt (2007)	High-throughput urine screening for Smith–Lemli–Opitz syndrome and cerebrotendinous xanthomatosis using negative electrospray tandem mass spectrometry
Salen *et al.* (1991)	Biochemical abnormalities in cerebrotendinous xanthomatosis
Salen and Steiner*.* (2017)	Epidemiology, diagnosis, and treatment of cerebrotendinous xanthomatosis (CTX)
Stelten *et al.* (2018)	Autism spectrum disorder: an early and frequent feature in cerebrotendinous xanthomatosis
Stelten *et al.* (2018)	Movement disorders in cerebrotendinous xanthomatosis
Vaz *et al.* (2017)	A newborn screening method for cerebrotendinous xanthomatosis using bile alcohol glucuronides and metabolite ratios
Verrips *et al.* (2000)	Presence of diarrhea and absence of tendon xanthomas in patients with cerebrotendinous xanthomatosis
Verrips *et al.* (1999)	Effect of simvastatin in addition to chenodeoxycholic acid in patients with cerebrotendinous xanthomatosis
Waterreus *et al.* (1987)	Cerebrotendinous xanthomatosis (CTX): a clinical survey of the patient population in The Netherlands
Yahalom *et al.* (2013)	Neurological outcome in cerebrotendinous xanthomatosis treated with chenodeoxycholic acid: early versus late diagnosis
Zubarioglu *et al.* (2017)	Early diagnosed cerebrotendinous xanthomatosis patients: clinical, neuroradiological characteristics and therapy results of a single center from Turkey

CTX: Cerebrotendinous xanthomatosis; EEG: Electroencephalogram; MR: Magnetic resonance.
