# Supplementary material for: Expert opinion on diagnosing, treating and managing patients with cerebrotendinous xanthomatosis (CTX): a modified Delphi study
Source: Orphanet J Rare Dis. 2021 Aug 6;16:353. doi: 10.1186/s13023-021-01980-5 (PMC8349076; doi:10.1186/s13023-021-01980-5)
Supplement: Supplementary file 6 — Additional file 6. Tables S4–S6 - Response distributions. [file 13023_2021_1980_MOESM6_ESM.pdf]

Response distributions

Supplementary Table 4. Response distribution for Likert scale questions

Question	Number of Panellists Selecting Likert Scale Option								Delphi Questionnaire Round
	Strongly Disagree	Disagree	Somewhat Disagree	Somewhat Agree	Agree	Strongly Agree	Do Not Wish to Answer	Insufficient Expertise	
*Please indicate symptoms that paediatric patients (aged <18 years old) present with, prior to a CTX diagnosis.**									
Chronic diarrhoea	0	0	1	0	2	7	0	0	Round 1
Bilateral juvenile cataracts	0	0	0	1	3	6	0	0	Round 1
Mental retardation (e.g. learning difficulties)†	0	0	0	0	5	5	0	0	Round 1
*Please indicate symptoms that adult patients (aged ≥18 years old) present with, prior to a CTX diagnosis.**									
Infantile-onset diarrhoea	1	0	1	1	2	5	0	0	Round 1
Childhood-onset cataracts	0	0	0	1	2	7	0	0	Round 1
Tendon xanthomas	0	0	1	0	3	6	0	0	Round 1
Psychiatric symptoms	0	0	0	1	5	4	0	0	Round 1
Peripheral neuropathy	0	0	0	3	4	3	0	0	Round 1
Cerebellar signs	0	0	0	0	5	5	0	0	Round 1
Pyramidal signs	0	0	0	1	6	3	0	0	Round 1

Question	Number of Panellists Selecting Likert Scale Option								Delphi Questionnaire Round
	Strongly Disagree	Disagree	Somewhat Disagree	Somewhat Agree	Agree	Strongly Agree	Do Not Wish to Answer	Insufficient Expertise	
All patients have elevated levels of serum cholestanol at the time of diagnosis.	0	0	0	0	2	8	0	0	Round 1
Brain MRI should be performed at the diagnosis stage as they can contribute to the diagnosis of CTX by revealing abnormally increased or decreased signals with characteristics distribution, but also to exclude other conditions.	1	0	0	2	3	4	0	0	Round 1
Measurement of serum cholestanol levels is the diagnostic marker of choice for CTX.	0	0	0	0	5	5	0	0	Round 1

Question	Number of Panellists Selecting Likert Scale Option								Delphi Questionnaire Round
	**Strongly Disagree**	**Disagree**	**Somewhat Disagree**	**Somewhat Agree**	**Agree**	**Strongly Agree**	**Do Not Wish to Answer**	**Insufficient Expertise**	
Movement disorders can be considered as late CTX manifestations, however, CTX should be considered in the differential diagnosis of movement disorders, particularly in case of an early onset and when associated with other neurological features and/or with systemic features.	0	0	1	2	3	4	0	0	Round 1
DBS testing is the optimal method for screening of CTX in newborns.	0	0	0	2	2	3	0	3	Round 1
CDCA is a lifetime replacement therapy.	0	0	0	0	4	6	0	0	Round 1

Question	Number of Panellists Selecting Likert Scale Option								Delphi Questionnaire Round
	Strongly Disagree	Disagree	Somewhat Disagree	Somewhat Agree	Agree	Strongly Agree	Do Not Wish to Answer	Insufficient Expertise	
The pathophysiological process in CTX patients may be reversed by CDCA, especially if treatment is initiated early in the disease process.	0	0	0	0	5	5	0	0	Round 1
Transcranial magnetic stimulation (TMS) is a useful tool for evaluating improvements in pyramidal function in patients receiving CDCA.	4	1	0	0	2	0	0	3	Round 1
Treatment adherence can be improved by providing CTX patients with support and intensive education.	0	0	0	1	5	4	0	0	Round 1

Question	Number of Panellists Selecting Likert Scale Option								Delphi Questionnaire Round
	**Strongly Disagree**	**Disagree**	**Somewhat Disagree**	**Somewhat Agree**	**Agree**	**Strongly Agree**	**Do Not Wish to Answer**	**Insufficient Expertise**	
Pre-marital genetic counselling should be recommended to high-risk populations e.g. patients of Israeli or Moroccan origin.	0	0	0	1	4	2	1	2	Round 1
Please indicate which of the below therapy options improves/stabilises prognosis in the majority of CTX patients.									
CDCA alone	0	0	0	0	1	9	0	0	Round 1
CDCA and HMG-CoA reductase inhibitor	0	0	2	0	1	6	0	1	Round 2
LDL apheresis	2	3	1	1	0	0	0	3	Round 2
Cholic acid alone	1	2	0	1	1	1	0	3	Round 3
Cholic acid and HMG-CoA reductase inhibitor	1	2	0	1	0	1	0	4	Round 3
Reducing plasma cholestanol concentrations slows down the progression of CTX.	0	0	1	2	5	2	0	0	Round 1

Question	Number of Panellists Selecting Likert Scale Option								Delphi Questionnaire Round
	Strongly Disagree	Disagree	Somewhat Disagree	Somewhat Agree	Agree	Strongly Agree	Do Not Wish to Answer	Insufficient Expertise	
CTX patients who start treatment after significant neurological pathology is established, have a worse prognosis compared to patients who started treatment as early as possible.	0	0	0	0	1	9	0	0	Round 1
CTX patients showing MRI evidence of cerebellar vacuolation should be monitored more strictly over time as it is considered a prognostic marker.	0	0	1	0	5	2	0	2	Round 1

Question	Number of Panellists Selecting Likert Scale Option								Delphi Questionnaire Round
	Strongly Disagree	Disagree	Somewhat Disagree	Somewhat Agree	Agree	Strongly Agree	Do Not Wish to Answer	Insufficient Expertise	
During the early stages of treatment, paediatric patients should be monitored for the types of symptoms listed below 1–2 times per year.									
Central and peripheral nervous system	0	0	0	0	2	7	0	1	Round 2
Ocular system	0	0	0	2	2	5	0	1	Round 2
Enterohepatic system	1	0	0	0	5	3	0	1	Round 2
Cognitive performance (e.g. learning difficulties)	0	0	0	0	2	7	0	1	Round 2
Cardiovascular system	0	3	2	0	3	1	0	0	Round 3
Skeletal system	0	0	1	2	4	2	0	0	Round 3
Pulmonary system	0	1	2	3	2	1	0	0	Round 3
During the early stages of treatment, adult patients should be monitored for the types of symptoms listed below once per year.									
Central and peripheral nervous system	0	0	0	0	1	9	0	0	Round 2
Ocular system	0	0	1	2	1	6	0	0	Round 2
Cardiovascular system	0	3	0	0	6	1	0	0	Round 2
Skeletal system	0	2	0	1	2	5	0	0	Round 2

Question	Number of Panellists Selecting Likert Scale Option								Delphi Questionnaire Round
	**Strongly Disagree**	**Disagree**	**Somewhat Disagree**	**Somewhat Agree**	**Agree**	**Strongly Agree**	**Do Not Wish to Answer**	**Insufficient Expertise**	
Enterohepatic system	0	1	0	1	4	4	0	0	Round 2
Cognitive performance (e.g. learning difficulties)	0	0	0	0	4	6	0	0	Round 2
Paediatric patients should undergo the types of tests listed below 1–2 times per year.									
Cholestanol plasma concentration	1	1	0	0	1	6	0	1	Round 2
Liver function tests	2	0	0	0	2	5	0	1	Round 2
Paediatric patients should undergo neurologic (and if necessary neuropsychologic evaluation) testing/examination twice per year.	0	0	1	1	3	4	0	1	Round 2
Adult patients should undergo the types of tests/examinations listed below once per year.									
Cholestanol plasma concentration	0	1	0	0	3	6	0	0	Round 2

Question	Strongly Disagree	Disagree	Somewhat Disagree	Somewhat Agree	Agree	Strongly Agree	Do Not Wish to Answer	Insufficient Expertise	Delphi Questionnaire Round
	Number of Panellists Selecting Likert Scale Option								
Neurologic (and if necessary neuropsychologic evaluation)	0	0	0	0	1	9	0	0	Round 2
Liver function tests	1	0	0	0	2	7	0	0	Round 2
Urinary bile alcohol concentration	0	1	1	2	3	0	0	2	Round 3
Brain MRI	0	3	1	3	2	0	0	0	Round 3
The following healthcare professionals are important in the diagnosis of paediatric patients with CTX.									
Neurologist	0	0	0	0	3	6	0	1	Round 2
Paediatrician/Metabolic specialist	0	0	0	1	1	7	0	1	Round 2
Geneticist	0	1	0	1	4	3	0	1	Round 2
Ophthalmologist	0	0	0	0	2	7	0	0	Round 3
Neuroradiologist	1	1	0	3	4	0	0	0	Round 3
Psychiatrist	0	0	3	3	2	1	0	0	Round 3
Orthopaedic surgeon	1	2	3	1	2	0	0	0	Round 3
Endocrinologist	1	5	2	0	0	1	0	0	Round 3
Gastroenterologist	0	1	2	3	2	1	0	0	Round 3

Question	Number of Panellists Selecting Likert Scale Option								Delphi Questionnaire Round
	Strongly Disagree	Disagree	Somewhat Disagree	Somewhat Agree	Agree	Strongly Agree	Do Not Wish to Answer	Insufficient Expertise	
The following healthcare professionals are important in the diagnosis of adult patients with CTX.									
Neurologist	0	0	0	0	1	9	0	0	Round 2
Metabolic specialist	0	1	0	1	1	7	0	0	Round 2
Geneticist	0	0	0	2	4	4	0	0	Round 2
Ophthalmologist	0	0	0	2	4	3	0	0	Round 3
Neuroradiologist	1	1	0	2	5	0	0	0	Round 3
Psychiatrist	0	0	0	3	3	3	0	0	Round 3
Orthopaedic surgeon	0	3	0	4	1	1	0	0	Round 3
Endocrinologist	0	5	0	2	1	1	0	0	Round 3
Gastroenterologist	0	2	2	2	2	1	0	0	Round 3
Cardiologist	0	5	1	1	1	1	0	0	Round 3
The following healthcare professionals should be involved in prescribing treatment to paediatric patients.									
Neurologist	0	0	0	2	3	4	0	1	Round 2
Neuroradiologist	4	3	1	1	0	0	0	1	Round 2
Paediatrician/Metabolic specialist	0	0	0	1	2	6	0	1	Round 2
Family doctor	3	4	0	0	2	0	0	1	Round 2
Endocrinologist	3	3	2	0	0	1	0	0	Round 3
Psychiatrist	2	2	2	1	2	0	0	0	Round 3

Question	Number of Panellists Selecting Likert Scale Option								Delphi Questionnaire Round
	Strongly Disagree	Disagree	Somewhat Disagree	Somewhat Agree	Agree	Strongly Agree	Do Not Wish to Answer	Insufficient Expertise	
The following healthcare professionals should be involved in prescribing treatment to adult patients with CTX.									
Neurologist	0	0	0	0	3	7	0	0	Round 2
Neuroradiologist	5	3	0	2	0	0	0	0	Round 2
Metabolic specialist	0	0	0	2	2	6	0	0	Round 2
Cardiologist	3	4	1	1	1	0	0	0	Round 2
Family doctor	3	4	0	1	2	0	0	0	Round 2
Ophthalmologist	3	4	2	0	0	1	0	0	Round 2
Endocrinologist	2	5	1	0	0	1	0	0	Round 3
Gastroenterologist	1	5	1	2	0	0	0	0	Round 3
Psychiatrist	2	2	0	3	2	0	0	0	Round 3
The following healthcare professionals should be involved in the follow-up of paediatric patients with CTX.									
Neurologist	0	0	0	1	2	6	0	1	Round 2
Paediatrician/Metabolic specialist	0	0	0	0	1	8	0	1	Round 2
Ophthalmologist	0	0	0	0	6	3	0	0	Round 3
Neuroradiologist	1	0	3	2	3	0	0	0	Round 3
Family doctor	0	0	0	4	4	1	0	0	Round 3
Endocrinologist	0	3	2	2	1	1	0	0	Round 3
Gastroenterologist	0	1	1	5	1	1	0	0	Round 3
Psychiatrist	0	1	0	4	3	1	0	0	Round 3

Question	Number of Panellists Selecting Likert Scale Option								Delphi Questionnaire Round
	Strongly Disagree	Disagree	Somewhat Disagree	Somewhat Agree	Agree	Strongly Agree	Do Not Wish to Answer	Insufficient Expertise	
The following healthcare professionals should be involved in the follow-up of adult patients with CTX.									
Neurologist	0	0	0	0	0	10	0	0	Round 2
Ophthalmologist	0	0	0	3	2	5	0	0	Round 2
Metabolic specialist	0	0	1	1	2	6	0	0	Round 2
Neuroradiologist	1	1	1	3	3	0	0	0	Round 3
Cardiologist	0	0	4	2	2	1	0	0	Round 3
Gastroenterologist	0	2	2	2	2	1	0	0	Round 3
Family doctor	0	0	0	4	3	2	0	0	Round 3
Endocrinologist	0	2	3	1	2	1	0	0	Round 3
Psychiatrist	0	1	1	2	3	2	0	0	Round 3
A specialist CTX centre/department should be visited once per year by:									
Adult patients with CTX	0	0	0	0	4	6	0	0	Round 2
Paediatric patients with CTX	0	0	0	1	4	4	0	1	Round 2
A local CTX centre/department should be visited twice per year by:									
Adult patients with CTX	0	0	1	0	5	4	0	0	Round 2
Paediatric patients with CTX	0	0	0	0	3	6	0	1	Round 2

Question	Number of Panellists Selecting Likert Scale Option								Delphi Questionnaire Round
	**Strongly Disagree**	**Disagree**	**Somewhat Disagree**	**Somewhat Agree**	**Agree**	**Strongly Agree**	**Do Not Wish to Answer**	**Insufficient Expertise**	
In patients with CTX, the absence of dentate nuclei signal alteration in brain MRI may be an indicator of better prognosis.	0	1	0	1	6	0	0	2	Round 2
Increased atrophy and/or signal alteration, identified through brain MRI examinations, may be present in patients who have deteriorating neurological symptoms.	1	0	0	1	6	1	0	1	Round 2

Question	Number of Panellists Selecting Likert Scale Option								Delphi Questionnaire Round
	**Strongly Disagree**	**Disagree**	**Somewhat Disagree**	**Somewhat Agree**	**Agree**	**Strongly Agree**	**Do Not Wish to Answer**	**Insufficient Expertise**	
Research indicates that treating CTX mothers with CDCA during pregnancy acts as an important means of protection against damage to the fetus and miscarriage.	0	0	1	1	3	1	0	3	Round 3
During the early stages of treatment, adult patients should be monitored for symptoms in the pulmonary system once per year.	0	1	2	2	2	1	0	1	Round 3
Paediatric patients should undergo testing for urinary bile alcohol concentrations once per year.	0	1	2	0	3	1	0	2	Round 3

Question	Number of Panellists Selecting Likert Scale Option								Delphi Questionnaire Round
	Strongly Disagree	Disagree	Somewhat Disagree	Somewhat Agree	Agree	Strongly Agree	Do Not Wish to Answer	Insufficient Expertise	
Paediatric patients should undergo brain MRI at the time of diagnosis, then once per year during follow-up.	1	0	4	3	1	0	0	0	Round 3
Disease progression in patients with CTX is better monitored using brain MRI compared with clinical evaluation alone.	1	3	0	3	2	0	0	0	Round 3
CDCA alone is a preferred first line treatment compared to CDCA and HMG-CoA reductase inhibitor for treating the underlying biochemical abnormalities in CTX.	0	0	0	2	1	6	0	0	Round 3

Question	Number of Panellists Selecting Likert Scale Option								Delphi Questionnaire Round
	Strongly Disagree	Disagree	Somewhat Disagree	Somewhat Agree	Agree	Strongly Agree	Do Not Wish to Answer	Insufficient Expertise	
There is a positive correlation between the progression of clinical and neuroradiological symptoms in patients with CTX.	0	0	0	4	4	0	0	1	Round 3
Brain MRI can be used to determine neurological stability in patients with CTX.	0	2	1	3	2	0	0	1	Round 3

A total of 10 panellists answered questions in Rounds 1 and 2, and 9 in Round 3. Questions achieving consensus (≥70% panellists agreeing/disagreeing with the statement) are shown for the round in which consensus was reached. Where questions did not achieve consensus throughout the study, the results are shown for Round 3. *Options that did not achieve consensus in Round 1 were rephrased as a proportion question in Round 2. Please refer to Table 5 for the rephrased questions and responses. [†]Phrased as in the original survey question; 'mental retardation' referred to as 'intellectual disability' in the text. CDCA: chenodeoxycholic acid; CTX: Cerebrotendinous xanthomatosis; DBS: dried bloodspot; HMG-CoA: 5-hydroxy-3-methylglutaryl-coenzyme A; LDL: low-density lipoprotein; MRI: magnetic resonance imaging; TMS: transcranial magnetic stimulation.

Supplementary Table 5. Response distribution for ranking questions

Question	Number of Panellists Selecting Ranking Position							Delphi Questionnaire Round
	1	2	3	4	5	Do Not Wish to Answer	Insufficient Expertise	
Please rank the following indicators in order of which has the greatest diagnostic value, when considering a CTX diagnosis (1=greatest diagnostic value; 5=least diagnostic value).								
CYP27A1 genetic mutation*	8	0	1	1	0	0	0	Round 2
An affected sibling	-	3	3	0	3	0	0	Round 3
Clinical signs and symptoms	-	2	3	4	0	0	0	Round 3
Biochemical pathogenesis	-	5	2	1	1	0	0	Round 3
Brain MRI findings	-	0	2	2	5	0	0	Round 3
Please rank the following tests/examinations in order of importance when confirming a CTX diagnosis (1=most important; 5=least important).								
Genetic testing alone	9	1	0	0	0	0	0	Round 2
Determination of serum cholestanol levels	2	8	0	0	0	0	0	Round 2
Detection of urinary bile alcohols	-	-	3	4	1	0	1	Round 3
Determination of plasma bile acids (mainly cholic acid and chenodeoxycholic acid)	-	-	3	5	1	0	0	Round 3
Conventional brain MRI	-	-	2	3	4	0	0	Round 3
Please rank the following factors in order of their impact on treatment outcomes in patients with CTX (1=greatest impact; 5=least impact).								
Age at diagnosis and treatment initiation	9	1	0	0	0	0	0	Round 2
Extent of neurological deterioration	2	8	0	0	0	0	0	
Cholestanol level at diagnosis	-	-	0	1	8	0	0	Round 3
Treatment compliance	-	-	6	2	1	0	0	Round 3

Question	Number of Panellists Selecting Ranking Position							Delphi Questionnaire Round
	1	2	3	4	5	Do Not Wish to Answer	Insufficient Expertise	
Characteristics of cerebellar signal abnormalities	-	-	3	6	0	0	0	Round 3
Please rank the following therapy options in order of their effectiveness for treating the underlying biochemical abnormalities in CTX (1=most effective; 5=least effective).								
CDCA alone	8	2	0	0	0	0	0	Round 2
LDL apheresis	0	1	1	0	5	0	3	Round 2
CDCA and HMG-CoA reductase inhibitor[†]	-	5	1	1	-	0	2	Round 3
Cholic acid alone	-	2	0	4	-	0	3	Round 3
Cholic acid and HMG-CoA reductase inhibitor	-	1	3	1	-	0	4	Round 3
Please indicate when the most beneficial time to start CTX treatment is by ranking the below options (1=most beneficial; 4=least beneficial).								
From birth following a positive newborn screening test for CTX	9	1	0	0	-	0	0	Round 2
Upon CTX diagnosis (with or without symptom onset)	2	8	0	0	-	0	0	Round 2
Upon symptom onset in patients diagnosed with CTX	1	0	9	0	-	0	0	Round 2
Upon presentation of neurological symptoms in patients diagnosed with CTX	0	1	0	9	-	0	0	Round 2

Question	Number of Panellists Selecting Ranking Position							Delphi Questionnaire Round
	1	2	3	4	5	Do Not Wish to Answer	Insufficient Expertise	
Please rank the following examinations and tests in order of their usefulness when monitoring paediatric patients receiving CTX treatment (1=most useful; 5=least useful).								
Cholestanol plasma concentration	7	2	0	0	0	0	1	Round 2
Neurologic examination (and if necessary neuropsychologic evaluation)	-	7	1	0	1	0	0	Round 3
Brain MRI	-	1	3	3	2	0	0	Round 3
Liver function tests	-	2	4	1	2	0	0	Round 3
Urinary bile alcohol concentration	-	1	1	4	2	0	1	Round 3
Please rank the following examinations and tests in order of their usefulness when monitoring adult patients receiving CTX treatment (1=most useful; 5=least useful).								
Cholestanol plasma concentration	7	3	0	0	0	0	0	Round 2
Neurologic examination (and if necessary neuropsychologic evaluation)	-	7	1	0	1	0	0	Round 3
Brain MRI	-	1	3	4	1	0	0	Round 3
Liver function tests	-	2	4	1	2	0	0	Round 3
Urinary bile alcohol concentration	-	1	0	4	3	0	1	Round 3

Question	Number of Panellists Selecting Ranking Position							Delphi Questionnaire Round
	1	2	3	4	5	Do Not Wish to Answer	Insufficient Expertise	
Please rank the remaining parameters in order of their usefulness for measuring treatment efficacy in patients with CTX (1=most useful; 5=least useful).								
Levels of serum cholestanol alone	2	2	2	2	1	0	0	Round 3
Clinical presentation/neurological examination	5	2	1	0	1	0	0	Round 3
Brain MRI	0	1	4	2	2	0	0	Round 3
Levels of urinary bile alcohols	1	0	2	3	2	0	1	Round 3
Electrophysiological examinations (e.g. electromyography, nerve conduction velocity, electroencephalography)	1	2	2	1	3	0	0	Round 3

A total of 10 panellists answered questions in Rounds 1 and 2, and 9 in Round 3. Question options achieving consensus (≥70% panellists selecting a particular ranking position for that option) are shown for the round in which consensus was reached. Where questions did not achieve consensus throughout the study, the results are shown for Round 3. In some cases, panellists assigned the same ranking position to multiple options. If consensus on a ranking position was achieved in Round 2, panellists were not asked to rank options in that position in Round 3. *Phrased as in the original survey question; 'genetic mutations' referred to as 'pathogenic variants' in the text. †Panellists came to consensus agreement about CDCA alone in Round 1 and CDCA in combination with HMG-CoA reductase inhibitors in Round 2, where CDCA alone was no longer included as an option. CDCA: chenodeoxycholic acid; CTX: Cerebrotendinous xanthomatosis; HMG-CoA: 5-hydroxy-3-methylglutaryl-coenzyme A; LDL: low-density lipoprotein; MRI: magnetic resonance imaging.

Supplementary Table 6. Response distribution for proportion questions

Question	Number of Panellists Selecting Proportion Option						Delphi Questionnaire Round
	0–24%	25–29%	50–74%	75–100%	Do Not Wish to Answer	Insufficient Expertise	
Please indicate the proportion of paediatric patients that present with the following symptoms, prior to a CTX diagnosis.							
Tendon xanthomas	8	1	0	0	0	0	Round 3
Early psychiatric symptoms (e.g. autism)	3	5	1	0	0	0	Round 3
Neonatal cholestatic jaundice	3	3	2	0	0	1	Round 3
Cerebellar system findings (e.g. ataxia symptoms and tremor)	3	2	3	1	0	0	Round 3
Epilepsy	3	5	1	0	0	0	Round 3
Peripheral neuropathy*	5	1	3	0	0	0	Round 3
Z-scores below the expected range for age in bone mineral density (BMD)*	4	1	1	0	0	3	Round 3
Please indicate the proportion of adult patients that present with the following symptoms, prior to a CTX diagnosis.							
Early-onset dementia	0	7	3	0	0	0	Round 2
Early-onset movement disorder (e.g. atypical parkinsonism)	4	4	0	1	0	0	Round 3
Epilepsy	5	3	1	0	0	0	Round 3

A total of 10 panellists answered questions in Rounds 1 and 2, and 9 in Round 3. Question options achieving consensus (≥70% panellists selecting a particular proportion for that option) are shown for the round in which consensus was reached. Where questions did not achieve consensus throughout the study, the results are shown for Round 3. In some cases, panellists selected the same proportion for different options. *In Round 2 these options were phrased in one option as 'Peripheral neuropathy where Z-scores

are below the expected range for age in bone mineral density (BMD)’, however, for scientific accuracy it was decided to split this into two options in Round 3. BMD: bone mineral density; CTX: Cerebrotendinous xanthomatosis.
